# Age-based spatial disparities of COVID-19 incidence rates in the United States counties

**DOI:** 10.1371/journal.pone.0286881

**Published:** 2023-06-08

**Authors:** Qian Huang

**Affiliations:** Center for Rural Health Research, College of Public Health, East Tennessee State University, Johnson City, Tennessee, United States of America; Villanova University, UNITED STATES

## Abstract

COVID-19 incidence disparities have been documented in the literature, but the different driving factors among age groups have yet to be explicitly explained. This study proposes a community-based COVID-19 spatial disparity model, considering different levels of geographic units (individual and community), various contextual variables, multiple COVID-19 outcomes, and different geographic contextual elements. The model assumes the existence of age nonstationarity effects on health determinants, suggesting that health effects of contextual variables vary among place and age groups. Based on this conceptual model and theory, the study selected 62 county-level variables for 1,748 U.S. counties during the pandemic, and created an Adjustable COVID-19 Potential Exposure Index (ACOVIDPEI) using principal component analysis (PCA). The validation was done with 71,521,009 COVID-19 patients in the U.S. from January 2020 through June 2022, with high incidence rates shifting from the Midwest, South Carolina, North Carolina, Arizona, and Tennessee to the West and East coasts. This study corroborates the age nonstationarity effect of health determinants on COVID-19 exposures. These results empirically identify the geographic disparities of COVID-19 incidence rates among age groups and provide the evidentiary guide for targeting pandemic recovery, mitigation, and preparedness in communities.

## 1. Introduction

The COVID-19 pandemic has sparked interest in age-specific manifestations of infection, but little is known about the relative severity of COVID-19 and its contributing factors between the extremes of age in the United States (U.S.) [1, 2]. One of the COVID-19 age-related features known is that children’s incidence and mortality rate was much less than in adults at the beginning of the pandemic [3]. Age distribution of deaths in the population under 65 years old is remarkably consistent across countries, and the case fatality rate is lowest among children age 5–9 years old, with an increase by age among the population 30 years and older [4]. Increased transmission of COVID-19 in older adults and limited access to adequate healthcare resulted in a higher burden in developing countries than in high-income countries [5]. In the U.S., only individuals age 20–49 sustained resurgent COVID-19 transmission reproduction over one, and at least 65% of infections originated from this age group as of October 2020 [6].

An essential step in studying age-specific manifestations of COVID-19 infection is to examine the different key factors driving the inequality of COVID-19 outcomes among different age groups. Currently, there is limited systematic analysis found in the literature of comprehensive factors that drive the severity differences of COVID-19 among different age groups. Previous studies on the association of health determinants with COVID-19 health disparities have focused on a single set of determinants. For example, the relationship between COVID-19 outcomes and contextual variables such as policies has been explored [7, 8]. An online survey of 4,676 U.S. adults age 18 and older suggested that people over 50 had less than half the predicted number of close contact behaviors than those under 30, therefore having fewer chances of contagion [9]. Similarly, measurable indicators of gender, education, ethnicity, employment status, number of children, immigrant status, social-economic status, and income have been used to demonstrate social determinants of health and social vulnerability [10, 11]. However, little attention has been paid to the differences in the compounding variable effects within different age groups. In addition, there is no conceptual model for COVID-19 health disparities among different age groups, and few works emphasize the role of social, behavioral, healthcare, environmental, and political determinants for each age group’s health.

To fill the research gap, this study aims to explore the nonstationarity effects of social, behavioral, environmental, healthcare access, and political contexts on COVID-19 outcomes across space and time among age groups. This requires an analysis of spatial variability in the COVID-19 outcomes among age groups and a breakdown of the driving factors behind such differences. This paper also proposes a community-based COVID-19 spatial disparity model and a new type of nonstationarity- age nonstationarity, which means the effects of contextual variables on health vary among age groups. The COVID-19 outcome in this study has been measured by the incidence rate (IR), defined as the rate of new cases of a disease in a population over a specified period [12]. A year-long interval has been chosen for differentiating the impacts of health determinants at the early (2020), middle (2021), and later (Jan-Jun 2022) stages of the pandemic. This temporal resolution provides a more stable environment for policy guidance throughout the various phases of the pandemic. Three questions guide this analysis:

1. What are the spatial and temporal distribution of COVID-19 IR in children (0–17 years), adults (18–64 years), and older adults (65 years and over) from Jan 2020 to Jun 2022?
2. How do COVID-19 IR age differences relate to pre-existing county conditions of social, behavioral, environmental, healthcare access, and political contexts in different years?
3. How to quantify the COVID-19 exposure risk in counties for different age groups?

To answer the first question, ArcGIS Pro showed the spatial and temporal distributions of 71,521,009 COVID-19 patients in the U.S. Over 60 variables representing pre-existing county conditions were included to examine the second question, and the main components were generated with principal component analysis (PCA). Furthermore, this study created an Adjustable COVID-19 Potential Exposure Index (ACOVIDPEI) to quantify the COVID-19 exposure risks at the county level by year. This work provides extended guidance for COVID-19 recovery, mitigation, and preparedness policymaking across age groups.

## 2. Nonstationarity and health disparity

There is often an assumption in health disparity research suggesting that the relationships between environmental factors and their health effects are stable over space and time. However, the impacts of health determinants may vary over space and time [13]. Eliminating health disparities is a goal endorsed by broad support. Public health research, to achieve this goal, usually adopts the perspective that social, economic, environmental, cultural, health behavioral, and political factors are the major contributors to unequal health status [14, 15]. Differential exposure to the virus, differential susceptibility to disease, and differential access to the health care system may also explain respiratory infection disparities [16].

### 2.1 Spatial, temporal, and age nonstationarity

Spatial nonstationarity and temporal nonstationarity have been confirmed in various COVID-19 studies [13, 17]. Understanding that the relationships between predictors and their health impacts are not stable over space and time is significant. Kim and Kwan [18] found that people’s mobility decreased between March- April 2020 in response to mobility restrictions but quickly bounced back to normal levels after the early stage, which might be due to “quarantine fatigue”. Another study in Hong Kong suggested that the changes in travel restrictions and quarantine requirements influenced the temporal distributions of spatial clusters of COVID-19 incidences over time [19].

Most discussions about vulnerabilities at age extremes are due to the complex medical needs, greater use of health care and support services in older adults, the immature immune system of infants, and the immune deterioration of older adults [20]. Despite their genetic and immune system differences, age has a determining effect on the relationship between social, behavioral, political, healthcare access, environmental factors, and their health. Therefore, age nonstationarity is proposed to indicate the varying effects of contextual variables on health among age groups. Taking this type of nonstationarity into account may lead to more accurate conclusions regarding the influence of various factors on the spread of COVID-19.

### 2.2 Health models

Detailed models of the potential causes of inequalities in disease burden were established to explore the risk factors [21, 22]. For example, one of the earliest and most widely known health disparity models is Andersen’s Behavioral Model and Access to Medical Care [23]. It portrayed the dynamic relationship between environment, population characteristics, health behavior, and health status outcomes. The CDC presented a four-level social-ecological model to better understand violence and the effect of potential prevention strategies [24], demonstrating the interplay between individual, relationship, community, and societal factors. All models previously described above have some merits in explaining the causes of health disparities. They have different emphases on genetics, socioeconomic characteristics, environments, behaviors, exposures, and scales. However, none of them cover the full dimensions of the factors driving COVID-19 outcome disparities, which include political, social, behavioral, environmental, and healthcare access. Moreover, none explain both vertical inequality (individuals or households) and horizontal inequality (age) for COVID-19 outcomes.

**Fig 1:**
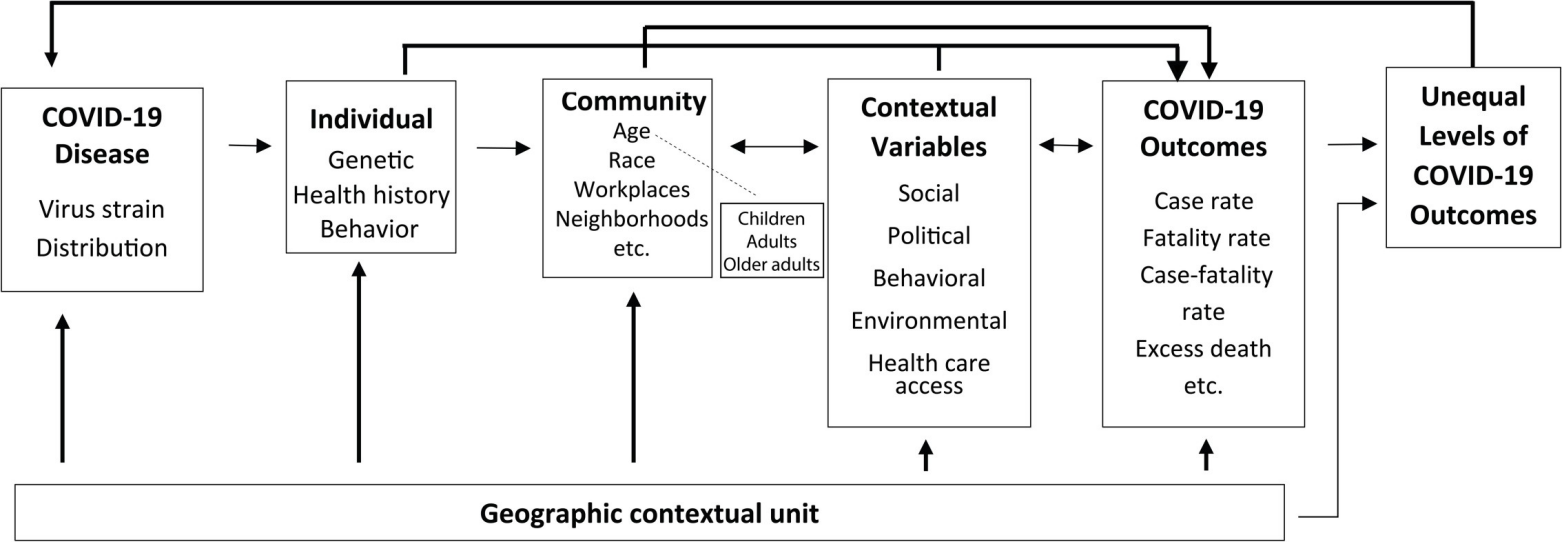
Community-based COVID-19 spatial disparity model.

To solve the issues, a community-based COVID-19 spatial disparity model has been proposed (Fig 1). The model begins with COVID-19 disease’s virus strain and its distribution, infecting the individual and community, then interacting with the contextual variables to yield COVID-19 outcomes. Genetic factors, health history, and behaviors influence individual outcomes along with contextual variables. Community typically examines different environmental settings, such as neighborhoods and workplaces, but in this model, the community is based on age groupings. An interaction is shown between the children, adults, and older adults, reflecting that every point of life helps define the individual life trajectory. Community by age group creates a direct impact on COVID-19 outcomes. Meanwhile, the contextual variables interact with individuals and the community (age) by changing social, political, behavioral, environmental, and healthcare access contexts to influence the distribution of COVID-19 outcomes. The COVID-19 outcomes can also impact contextual variables, for example, changing healthcare access by increasing telehealth services and mobile clinics. Moreover, the geographic contextual unit plays a vital role throughout the process. Similarly, the unequal levels of COVID-19 outcomes are a product of the geographic contextual units and contextual variables. The unequal levels of COVID-19 outcomes may change its distribution when new virus variants appear.

### 2.3 Drivers of COVID-19 disparities

#### 2.3.1 Political affiliation and intervention

Political party affiliation influences COVID-19 exposure and vaccination [25]. Studies showed that the COVID-19 pandemic has killed more Republican voters than Democratic voters since more Republicans opposed public health measures such as mask mandates and vaccination requirements [26]. Research also confirmed that physical distancing, mask mandates, stay-at-home orders, and business and school closures lower COVID-19 morbidity and mortalities [27]. For example, face mask mandate violation has been shown to be significantly correlated with COVID-19 death rates in New York City [28]. In the U.S., mitigation efforts decreased the spread of COVID-19 cases in some communities [8], even though not all groups had the capacity to take voluntary mitigative action.

#### 2.3.2 Social economic factors

Studies demonstrated that the potential of existing inequalities due to social factors such as gender, race, poverty, employment status, education level, occupation, discrimination, and bias contributes to a greater liability of morbidity and mortality from COVID-19, thereby exacerbating health disparities [29]. Karmakar et al. [30] confirmed that an increase of 0.1 points in the Social Vulnerability Index (SVI) score was associated with a 14.3% increase in the COVID-19 incidence rate. Moreover, African American individuals have a long history of structural racism that impedes access to healthcare resources and services, resulting in growing health inequities [31]. The workers in protective service occupations (e.g., police officers, firefighters), office and administrative support occupations (e.g., couriers), healthcare sectors, community and social services occupations, and construction occupations have a higher exposure risk to infections [32]. Poverty is another historical factor in preventing adequate healthcare. For example, a study confirmed that poor neighborhoods in New York City had fewer people tested for COVID-19 [33].

#### 2.3.3 Behavior, perception, and comorbidity

Behavioral determinants of health include health behaviors, comorbidities, prior experiences, and health perceptions. Behaviors that changed due to COVID-19 were decreased physical activity, sleep, diet behaviors, increased handwashing; wearing a face mask; keeping a distance from others; and working or attending school from home [34, 35]. An online survey exploring the influence of the COVID-19 pandemic reported that United Kingdom participants had the lowest levels of physical health and the highest increase in weight, while Latin American responders were most affected by emotions [35]. Studies also showed that male adolescents in Poland, Norway, and Jordan, and young adult men in Switzerland were less likely to report protective handwashing compare with females [34, 36, 37].

Perception of the risk plays a vital role in COVID-19 outcomes. Perceptions of COVID-19 risk positively affect their understanding of and participation in protective behaviors such as social distancing and handwashing [38, 39]. Abdelrahman [40] found that, in Qatar, the more highly responders rated the danger of COVID-19, the more likely they were to socially distance themselves. Similarly, anxiety regarding COVID-19 and fear of death of adults in Portugal significantly predicted protective behaviors, influenced by risk perceptions [41].

COVID-19 patients with comorbidities such as obesity, cardiovascular diseases (CVD), hypertension, diabetes, chronic obstructive pulmonary disease (COPD), malignancy, renal diseases, and HIV have a higher risk of severe illness and hospitalization [42]. For instance, there were 47.6% of people with obesity (BMI ≥ 30 kg/m^2^) became infected with COVID-19, and 68.6% of them were critically ill, receiving ventilation, early in the pandemic [43]. Patients under 60 with obesity were 1.8 and 2 times more likely to be admitted to acute and critical care [44]. Likewise, patients over 60 with other comorbidities like chronic respiratory disease and diabetes were also at a higher risk of COVID-19 infection [42].

#### 2.3.4 Environmental components

Substantial research has demonstrated that the severity and time of expansion of most diseases are caused by interactions between genetic, behavioral risk, and environmental factors rather than “bad genes” [45]. The COVID-19 pandemic has shed more light on the critical role of local parks and recreation as they provide spaces to support physical and mental health [46]. Meteorological and geophysical hazards interacted with COVID-19 exposures in many regions. For instance, the daily new infection rate showed an apparent increase following the Zagreb earthquake on March 20^th^, 2020, within the COVID-19 incubation time range [47]. The availability of safe water, clean air, and sanitation is essential for health and COVID-19 prevention. Moreover, environmental exposures to air pollution and toxic substances are essential contributors to COVID-19 [48]. A report from Harvard University suggested that long-term exposure to PM2.5 can lead to an increase in the COVID-19 death rate in the U.S. [49].

#### 2.3.5 Healthcare provider and access

Studies supported that access to the healthcare system, such as hospitals, emergency departments, ventilators, and telemedicine, has played a crucial role during the COVID-19 pandemic [50, 51]. Azar et al. [50] observed that non-Hispanic Black patients had 2.7 times the odds of hospitalization compared with non-Hispanic White patients, attributed barriers to timely healthcare access. Moreover, Moorthy and Sankar [52] surveyed Black Asian and minority ethnic (BAME) group’s perceptions and beliefs for the disproportionate death, and reported that lack of personal protective equipment (PPE) (58.5%) and lack of testing (46.5%) were their top reasons. The existing inequalities in healthcare access contribute to a greater difference in the COVID-19 burden and create obstacles to reduce health disparities.

#### 2.3.6 Age

Age has contrasting effects on respiratory infectious diseases. Studies found that children have milder COVID-19 clinical symptoms and fewer laboratory and radiologic abnormalities, while older adults have a higher mortality rate than other age groups early in the pandemic [53, 54]. However, this is not always the case. During June-August 2020, SARS-CoV-2 infection was highest in individuals 20–29 years, who accounted for over 20% of all confirmed cases [55]. In June 2020, increases in the percentage of positive COVID-19 test results among adults 20–39 years preceded increases among adults over 60 years by 4–15 days across the southern U.S. [55]. As of August 2022, the death rate was 140 times higher in the 75–84 age group and 340 times higher in those 85 years and older, compared to adults 19–29 years old [56]. COVID-19 racial disparities were substantial before April and generally decreased later in 2020 among persons under 25 in 15 U.S. states and the District of Columbia [57]. Black COVID-19 patients age 18–49 were more than twice as likely to be hospitalized from the emergency department (ED) as non-Black patients, while older groups (50–63, 65+) were not race-related to this outcome [58].

## 3. Data and methodology

### 3.1 COVID-19 incidence rates disparities

The COVID-19 incidence by age group data has been collected from the Centers for Disease Control and Prevention (CDC) [59]. The case surveillance dataset has patient-level COVID-19 cases shared with CDC and includes demographics, county and state of residence, any exposure history, disease severity and outcomes, and underlying medical conditions. The study period is from the beginning of January 2020 through the end of June 2022. The analysis did not include cases reported from U.S. territories, including those from Guam, the Northern Mariana Islands, Puerto Rico, and the U.S. Virgin Islands. The death information was excluded in this study due to the data discrepancy. The patients are grouped into children (0–17 years old), adults (18–64 years old), and older adults (over 65 years old) based on data source specified categories and aggregated to their residence counties. All data is fully anonymized and publicly available to all without restrictions, and no institutional review is needed [59]. Data cells are suppressed for low frequency (<11 COVID-19 case records with a given value) to prevent identifying people. Suppression includes low-frequency combinations of case month, geographic and demographic characteristics [59]. Therefore, age-identified COVID-19 incidence data were available in 1,748 counties. To examine the COVID-19 outcome disparities, incidences and incidence rates (IR) have been compared among age groups and pandemic years and mapped in ArcGIS Pro 2.8.0. COVID-19 IR by age group is calculated as follows in this study:

$$\frac{\;number\;of\;new\;children\;cases\;during\;a\;specified\;time\;period\;}{\;size\;of\;children\;population\;at\;start\;of\;time\;period\;}*100,000=\text{IR}\;\text{for}\;\text{children}$$ (1)

$$\frac{\;number\;of\;new\;adults\;cases\;during\;a\;specified\;time\;period}{\;size\;of\;adults\;population\;at\;start\;of\;time\;period\;}*100,000=IR\;\text{for}\;\text{adults}$$ (2)

$$\frac{number\;of\;new\;older\;adult\;cases\;during\;a\;specified\;time\;period\;}{\;size\;of\;older\;adult\;population\;at\;start\;of\;time\;period\;}*100,000=\text{IR}\;\text{for}\;\text{older}\;\text{adults}$$ (3)

For example, there were 88,982 children residing in Richland County, South Carolina, with 2,461 of them got affected by COVID-19 in 2020. Therefore,

IRforchildreninRichlandCountyin2020=2461/88982*100,000=2765.73 (4)

### 3.2 Determinants of health and statistical analysis

To examine the age nonstationarity impacts of health determinants on COVID-19 exposure risks, the study collected over 60 variables representing pre-existing conditions in counties with available incidence rates. Specifically, 62 variables were gathered across 1747 counties in 2020. For the following years, 2021 and 2022, 61 variables (FEMA Federal Support excluded) were compiled for 1739 counties and 1669 counties, respectively. Missing county data were substituted with state or national averages. Among all variables, 57 are static, and 5 are time series data. S1 Table summarizes all spatial data inputs. Listed below are detailed explanations of the input data and data manipulations.

#### 3.2.1 Political affiliation and intervention data

In order to measure the COVID-19 policy intervention efforts, the study incorporated data from four sources (S1 Table). COVID-19 policies, which are time series data, encompass emergency declarations, mask mandates, daycare and business closures, and stay-at-home orders from early 2020 to June 2022. A binary system was used to indicate the presence (1) or absence (0) of mandates for county-specific and statewide datasets on four mitigation measures. If there was a statewide mandate for any prevention efforts, all counties in the state received one score for the presence and zero if they opted out. Scores for county-ordered and statewide efforts ranged from 0–4, respectively. The impact was assessed in combination (0–8 range) for policies [8] in each year based on the policy implementation duration. Political affiliation was represented by the percentage of voting Democrat and obtained from MIT 2020 Election Data and Science as static data.

#### 3.2.2 Social economic data

Eighteen variables were selected to represent the pre-existing county socioeconomic conditions, including poverty, median household income, renter, married population, fender, race, language, female-headed households, households with children, education attainment, population density, and housing units with no car from American Community Survey 5-Year Data (2016–2020). Additionally, unemployment rates, income inequality, population growth, health insurance, healthcare-related occupation, and population with disabilities were sourced from other data sources (S1 Table). All socioeconomic data collected are static in nature.

#### 3.2.3 Behavior, perception, and comorbidity data

The data on behavior, perception and comorbidity consists of fifteen static variables alongside one time series variable. Publicly available COVID-19 vaccination rates were downloaded from CDC [60] in August 2022. The time series vaccination rates were the cumulative percentage of people who completed the primary series (receiving a second dose of a two-dose vaccine or one dose of a single-dose vaccine) in 2020, 2021, and 2022. The comorbidity data include hypertension, cardiovascular disease, stroke, mental health, asthma, HIV, diabetes, depression, obesity, low birth weight, and cancer from CDC PLACES [61] and BRFSS County Health Rank [62]. Religious affiliation was retrieved from the US Religious Census, while alcohol consumption, physical inactivity, and social associations were acquired from BRFSS County Health Rank [62].

#### 3.2.4 Environmental data

The COVID-19 pandemic has emphasized the vital role of local parks, recreation, air quality, mobility, and crime rates as they provide spaces to support physical and mental health. Thirteen variables were included, with two of them being time series data. Workplace mobility change and grocery and pharmacy mobility data were collected from Google COVID-19 community mobility reports [63], with average daily changes from baseline calculated each year. Additional variables were included, such as access to parks, recreation facilities, food environment index, environmental hazards, Ozone days, and violent crime (S1 Table).

#### 3.2.5 Healthcare provider and access

Healthcare provider and access encompass standardized numbers of healthcare providers, facilities, services, and federal support. The healthcare provider and facility data were obtained from the Health Resources & Services Administration (HRSA) Area Health Resources Files [64], and standardized per 100,000 population. FEMA obligated essential resources throughout the nation during the initial stages of the COVID-19 pandemic, rendering it a time series variable only applicable in 2020.

#### 3.2.6 Statistical analysis

Principal components analysis (PCA) reduced the data and generated the determinants of health main components each year. Subsequently, Pearson correlations were conducted between COVID-19 IR by age group (children, adults, older adults, and overall, respectively) and the health components for the years 2020 (early stage), 2021 (middle stage), and 2022 as of June (later stage). The statistical analyses were performed in IBM SPSS Statistics 28.

### 3.3 Build the Adjustable COVID-19 Potential Exposure Index (ACOVIDPEI)

To further understand the potential exposure disparities, this study developed an Adjustable COVID-19 Potential Exposure Index (ACOVIDPEI) based on each component’s effect on COVID-19 incidence for different age groups in different periods: a positive (+) increases the chance of getting infected, while a negative (-) decreases the chance of infection. The factor scores then could be placed in an additive model based on their cardinalities to composite the ACOVIDPEI score for each county for age communities. The study made no priori assumption about the importance of each factor in the overall sum. The composition of ACOVIDPEI is adjustable since the cardinality of the components can be modified based on their impact on community groups.

An example index for older adults in 2020 has been created. To determine the most and least COVID-19 risk, the ACOVIDPEI-Older Adults scores were mapped using a three-category classification based on standard deviations from the mean ranging from -1 on the lower end to +1 on the upper end. To validate the index’s performance, a Pearson correlation was conducted between the COVID-19 IR in 2020 of older adults and the index score. These analyses were done in IBM SPSS Statistics 28 and ArcGIS Pro 2.8.0.

## 4. Results

### 4.1 Spatial and temporal distribution of COVID-19 individual exposure

As of June 2022, 77,544,202 COVID-19 patients have been recorded, 1.76% of these (1,361,571 patients) have no age information, and 6.01% of the data (4,661,622) have no residence records. After excluding the cases with missing age information, county of residence, and infection date, a total of 71,521,009 patients were included in this study, located in 1,748 U.S. counties. The data availability varies among age groups and pandemic years (Table 1). Of the total counties in the U.S., 82.4% (961 out of 1,166) of the metro counties and 39.8% (787 out of 1,976) of the nonmetro counties based on the USDA Rural-Urban Continuum Codes 2013 (RUCC) designation were included in this study. The overall IR of children from Jan 2020 to Jun 2022 was similar to that in the older adults (children: 17,873.06 vs. older adults: 16,874.04 per 100,000 population), while the IR in the adult group was around 1.5 times higher than other groups.

**Table 1:** Descriptive statistics of COVID-19 incidence data by year and age group from Jan 2020 to June 2022.

Year		Children	Adults	Older Adults	Missing	Total
**2020**	# of counties	1,732	1,747	1,745		
	Incidences	1,874,704	13,343,773	2,513,745	1,450,924	19,183,146
	Incidence rates	2694.23	6974.59	5116.46		
**2021**	# of counties	1,736	1,737	1,735		
	Incidences	5,730,335	21,445,589	3,149,358	2,330,653	32,655,935
	Incidence rates	8253.56	11255.43	6439.43		
**2022 (as of June 30)**	# of counties	1,656	1,663	1,653		
	Incidences	4,860,561	15,973,079	2,629,865	2,241,616	25,705,121
	Incidence rates	7178.68	8538	5508.14		
**Overall**	Incidences	12,465,600	50,762,441	8,292,968	6,023,193	77,544,202
	Incidence rates	17873.06	26530.57	16874.04		

**Fig 2:**
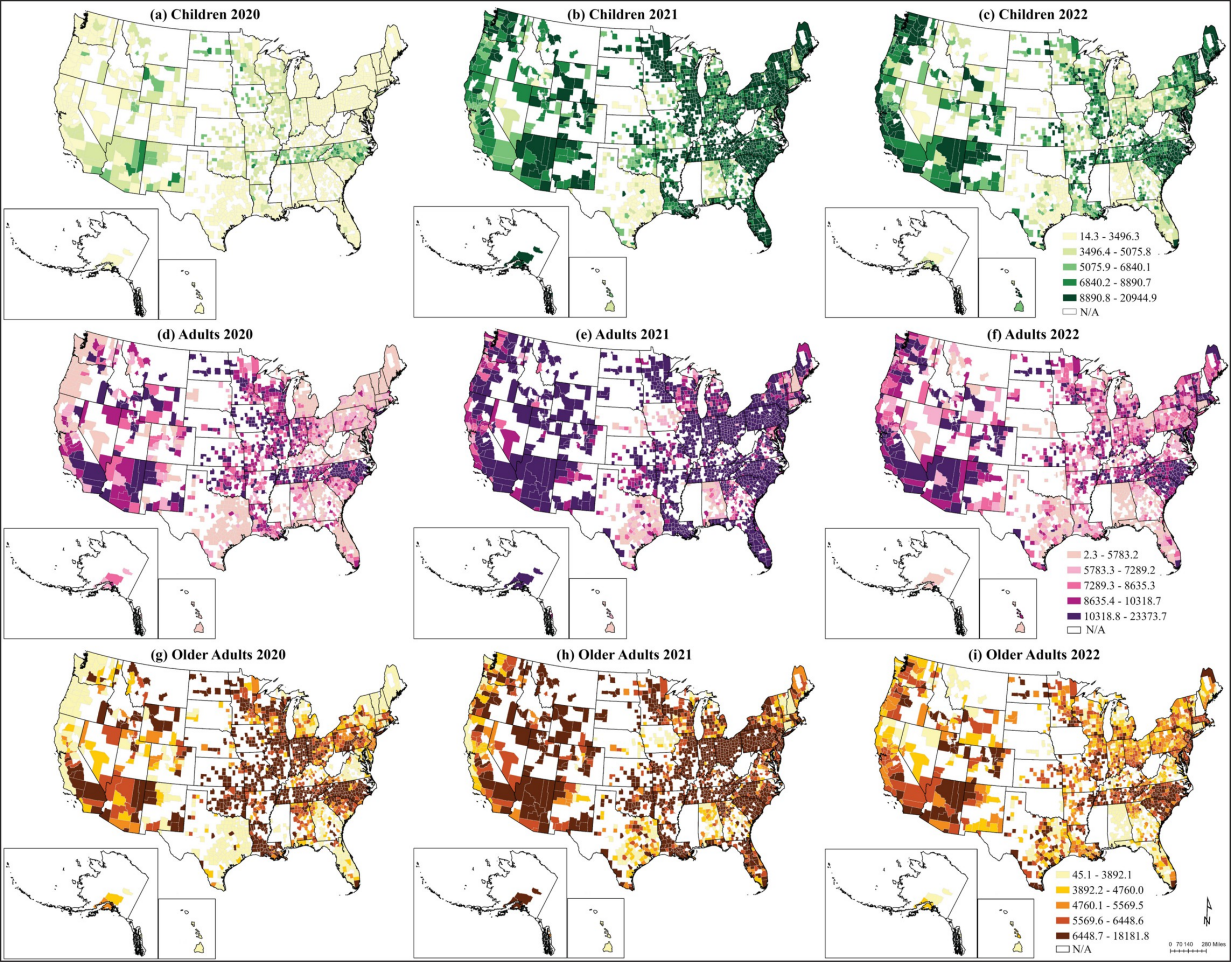
County-level COVID-19 IR by year (2020, 2021, and 2022 as of June 30) for children, adults, and older adults. County and state boundaries are retrieved from the U.S. Census Bureau (https://www.census.gov/geographies/mapping-files/time-series/geo/carto-boundary-file.html). The shapefiles are released under the CC BY 4.0 license.

In 2020, the COVID-19 IR of adults in those 1,747 counties was 2.6 times higher than children and 1.4 times higher than older adults. Geographically, the county-level distribution of IR varies across the states but the pattern difference between age groups was not prominent (Fig 2A 2D and 2G). The top 20% of the IR (> 3,970.4 cases per 100,000 population for children, >10,175.1 for adults, and >7,949.3 for older adults) were located in the Midwest (Minnesota, Wisconsin, North Dakota, Iowa), the Southeast (North Carolina, Tennessee), and the Southwest states (Arizona and California) (Fig 2). The lowest 20% of the IR appeared in the Northeast and Texas (<1,384 for children, <4,321.8 for adults, and <2,936.6 for older adults).

In 2021, IR increased among all age groups: IR in children was 3 times higher than in 2020, while in adults was 1.6 times and in older adults was 1.3 times higher than in 2021 (Table 1). However, the gap in COVID-19 IR between adults and children reduced to 1.4 times, possibly due to a significant increase in infections in the children group. The gap between adults and older adults expanded 1.7 times (adults: 11,255.43 vs. older adults: 6,439.43 per 100,000 population). This indicates a relatively slower increase in COVID-19 IR in the older adult group. The highest top 20% of the IR for children shifted to Northeast, Carolinas, Tennessee, Louisiana, Florida, and Alaska (>11,189.7 per 100,000 population) (Fig 2B). The lowest 20% of the IR for children were in Texas, Nebraska, and Alabama (< 5,777 incidences per 100,000 population). For adults, over 21 million cases were reported with the top quantile of IR (>13,973.2 per 100,000 population) in Arizona, Minnesota, Illinois, Indiana, Kentucky, Louisiana, Florida, and Alaska (Fig 2E). The older adult group showed a slightly different pattern from other age groups. The highest IR for older adults were clustered in Pennsylvania, Ohio, Indiana, Arizona, Alaska, and South Carolina (>8,412.7 cases per 100,000 population) (Fig 2H).

As of June 30^th^, 2022, over 4.8 million child patients were reported for 2022, with an IR of 7,178.68 cases per 100,000 population. At the same time, the COVID-19 IR for adults was 8,538, and for older adults was 5,508.14 per 100,000 population. The top 20% of the IR for all three age groups shifted to Carolinas, Tennessee, Arizona, Maine, west coast, and east coast. The lowest IR were in Nebraska, Kentucky, Alabama, and some counties in Texas (Fig 2C, 2F and 2I).

### 4.2 Role of pre-existing county conditions in explaining COVID-19 IR spatial disparities

PCA was used to identify contextual variables for explaining COVID-19 outcomes each year. In 2020, thirteen components were produced from 62 contextual variables on health determinants to differentiate U.S. counties. The PCA explained 69% of the variance among all counties. Fourteen components were produced in 2021 and 2022, explaining 72% of the variance. The component names were decided by the variables loaded highest in the components (usually >0.7 or <-0.7), and the top variables were included in Tables 2–4. The full details are listed in S2–S4 Tables.

**Table 2:** Dimensions of COVID-19 related determinants of health in 2020.

Component	Name	% Variance Explained	Dominant Variables	Component Loading
**1**	Comorbidities and social status	21.889	Stroke	0.935
			Cardiovascular diseases (CVD)	0.933
			Mental health	0.865
			Below poverty	0.863
**2**	Healthcare providers	8.293	Primary care physicians	0.853
			Internal medical doctors (MDs)	0.842
			Pediatrician	0.798
**3**	Race and chronic diseases	8.140	Race—Non-White	0.758
			Depression	-0.668
			Cancer (excluding skin cancer)	-0.748
**4**	Households without children	4.956	Households with children	-0.671
**5**	School and healthcare access	4.285	School	0.705
			Hospitals	0.607
**6**	Urbanism	3.892	Population density	0.848
			Housing units with no car	0.831
**7**	Natural amenity	3.416	Natural amenities scale	0.807
**8**	Religion	3.296	Religious affiliation	0.700
**9**	Air quality	2.972	Particulate matter days	0.758
			Ozone days	0.715
**10**	Nursing homes	2.102	Nursing homes admissions	0.752
**11**	Environmental hazards	2.063	Environmental hazards	0.694
**12**	Vaccinations	1.979	COVID-19 Vaccination Rates	0.577
**13**	Mental health centers	1.921	Mental health centers	0.748
	*Total Variance Explained*	*69*.*206*		

**Table 3:** Dimensions of COVID-19 related determinants of health in 2021.

Component	Name	% Variance Explained	Dominant Variables	Component Loading
**1**	Comorbidities and social status	22.480	Cardiovascular diseases (CVD)	0.936
			Stroke	0.929
			Mental health	0.877
			Physical inactivity	0.872
**2**	Race and political affiliation	9.161	Race—Non-White	0.738
			Democratic voters	0.681
			Married population	-0.746
**3**	Healthcare providers	6.810	Primary care physicians	0.813
			Internal medical doctors (MDs)	0.804
			Pediatrician	0.764
**4**	School and healthcare access	4.696	Hospitals	0.707
			School	0.692
**5**	Natural amenity	3.901	Natural amenities scale	0.848
**6**	Occupation and language	3.704	Healthcare related occupation	0.635
			Language/ability to speak English	-0.526
**7**	Mobility	3.571	Grocery and pharmacy mobility	0.631
**8**	Urbanism	3.506	Population density	0.876
			Housing units with no car	0.828
**9**	Air quality	2.749	Particulate matter days	0.790
			Ozone days	0.718
**10**	Policies and liquor stores	2.600	Policies	0.644
			Liquor store density	0.631
**11**	Asthma	2.566	Asthma	-0.549
**12**	Nursing homes	2.040	Nursing homes admissions	0.815
**13**	Environmental hazards	1.984	Environmental hazards	0.766
**14**	Mental health centers	1.885	Mental health centers	0.812
	*Total Variance Explained*	*71*.*654*		

**Table 4:** Dimensions of COVID-19 related determinants of health in 2022 as of June 30.

Component	Name	% Variance Explained	Dominant Variables	Component Loading
**1**	Comorbidities and social status	22.242	Cardiovascular diseases (CVD)	0.948
			Stroke	0.912
			Mental health	0.895
			Physical inactivity	0.860
**2**	Race and chronic diseases	9.017	Race—Non-White	0.830
			HIV	0.706
			Cancer (excluding skin cancer)	-0.671
**3**	Healthcare providers	7.679	Primary care physicians	0.839
			Internal medical doctors (MDs)	0.811
			Pediatrician	0.769
**4**	Household occupation and children	4.395	Healthcare related occupation	0.642
			Households with children	-0.574
**5**	School and healthcare access	4.282	School	0.706
			Hospitals	0.632
**6**	Urbanism	3.583	Population density	0.866
			Housing units with no car	0.827
**7**	Natural amenity	3.540	Natural amenities scale	0.794
**8**	Air quality	2.978	Particulate matter days	0.806
			Ozone days	0.742
**9**	Liquor stores	2.806	Liquor store density	0.677
**10**	Religions	2.762	Religious affiliation	0.721
**11**	Policies	2.353	Policies	-0.709
**12**	Nursing homes	2.147	Nursing homes admissions	0.839
**13**	Mental health centers	1.944	Mental health centers	0.781
**14**	Environmental hazards	1.895	Environmental hazards	0.813
	*Total Variance Explained*	*71*.*622*		

Most of the main components are similar in the three pairs of PCAs. For example, all of the first factors identified in three PCAs are comorbidities and social status of counties as measured by the age-adjusted prevalence of diseases such as stroke, CVD, mental health, physical inactivity, and social status such as poverty. It explains around 22% of the variance. The second and third factors identified healthcare providers, race, chronic diseases, and political affiliations, which present 16% of the variance among counties. Moreover, school and healthcare access, urbanism, natural amenity, air quality, nursing homes, environmental hazards, and mental health centers are generated in all PCAs.

Nevertheless, there are some nuances in other key components. For example, households with children and religion loaded as main components in the 2020 and 2022 PCAs but not in 2021. Vaccination was only a significant component in 2020, while the mobility change component was only loaded in 2021. In addition, PCAs identified policies in the middle and later stages of the pandemic, but not in the early stages. Moreover, liquor store density was found as the 9^th^ main component in 2022, explaining 2.8% of the variability in county contextual attributes (Table 4).

To explore the driving factors of the different spatial distribution of COVID-19 IR in children (0–17 years), adults (18–64 years), and older adults (65 years and over) from 2020 to 2022, Pearson correlations were conducted between IR across counties and determinants of health among pandemic years and age groups (Table 5). Almost all relationships were significant, therefore, only the correlation coefficients higher than 0.1 or lower than -0.1 at p<0.01 level were reported below.

**Table 5:** Correlations between COVID-19 IR and determinants of health.

Year	Factors	All	Children	Adults	Older Adults
2020	# of counties	1747	1732	1747	1745
	Factor 1	.105^**^	.054^*^	.059^*^	.163^**^
	Factor 2	-.050^*^	0.020	-.049^*^	-0.045
	Factor 3	-0.025	-0.025	0.010	-.105^**^
	Factor 4	-.086^**^	-.094^**^	-.125^**^	-0.034
	Factor 5	.204^**^	.097^**^	.226^**^	.159^**^
	Factor 6	-0.024	-.056^*^	-0.031	0.039
	Factor 7	-.199^**^	-.056^*^	-.188^**^	-.325^**^
	Factor 8	.256^**^	.251^**^	.230^**^	.257^**^
	Factor 9	.055^*^	0.010	.082^**^	.054^*^
	Factor 10	-.062^**^	-.077^**^	-.053^*^	-0.045
	Factor 11	.091^**^	.082^**^	.088^**^	.109^**^
	Factor 12	-0.017	0.018	-0.014	-0.024
	Factor 13	-.059^*^	-.089^**^	-.080^**^	-.055^*^
2021	# of counties	1739	1736	1737	1735
	Factor 1	.124^**^	-0.013	.049^*^	.154^**^
	Factor 2	-.091^**^	-0.024	-.066^**^	-.115^**^
	Factor 3	-0.011	.048^*^	-0.020	-.052^*^
	Factor 4	-0.042	-.077^**^	-0.031	0.035
	Factor 5	-.133^**^	-.067^**^	-.105^**^	-.182^**^
	Factor 6	.127^**^	.291^**^	.108^**^	-0.012
	Factor 7	.071^**^	.114^**^	.061^*^	0.013
	Factor 8	.085^**^	.078^**^	.081^**^	0.029
	Factor 9	-0.002	-.084^**^	0.030	.051^*^
	Factor 10	.149^**^	.186^**^	.090^**^	-0.008
	Factor 11	-.202^**^	-.192^**^	-.218^**^	-.192^**^
	Factor 12	-0.005	-0.044	0.015	0.035
	Factor 13	.114^**^	0.029	.112^**^	.098^**^
	Factor 14	.131^**^	.052^*^	.070^**^	.092^**^
2022	# of counties	1668	1656	1668	1653
	Factor 1	-0.009	-.094^**^	-.087^**^	-0.004
	Factor 2	.086^**^	.146^**^	.053^*^	.076^**^
	Factor 3	.180^**^	.240^**^	.153^**^	.167^**^
	Factor 4	.054^*^	.135^**^	0.043	-0.043
	Factor 5	-.091^**^	-.115^**^	-0.033	-0.035
	Factor 6	-0.001	0.018	-0.014	0.002
	Factor 7	0.015	.085^**^	-0.011	-.059^*^
	Factor 8	.078^**^	0.040	.083^**^	.106^**^
	Factor 9	.145^**^	.178^**^	.103^**^	-0.003
	Factor 10	-.142^**^	-.221^**^	-.176^**^	-0.047
	Factor 11	-.064^**^	-.076^**^	-.100^**^	-.079^**^
	Factor 12	-.102^**^	-.123^**^	-.068^**^	-.078^**^
	Factor 13	.069^**^	-0.008	-0.011	0.012
	Factor 14	-0.038	-.076^**^	-0.021	-0.028

**. *p*< 0.01 (2-tailed)

*. *p*<0.05 (2-tailed)

#### 4.2.1 All ages

In 2020, high IR was significantly associated with high comorbidities and low social status, high access to school and healthcare, a high percentage of religious affiliations, and worse natural amenities. This confirmed that large religious gatherings, high access to school, and insufficient natural amenities were important drivers of COVID-19 infection at the early stage of the pandemic [65–67]. Interestingly, religion became negatively associated with the COVID-19 incidence rate in 2022. Factors- occupation and language, policies and liquor stores, asthma, environmental hazards, and mental health centers became more significant in 2021. This emphasized the higher infection rates among healthcare-related workers and the White population, locations with short-term COVID-19 mitigation measures, and the significant side effects of alcohol and air pollution [25]. In 2022, as of June 30, Factor 3-Healthcare providers became positively significant, suggesting more exposure contacts in sufficient healthcare areas. Nursing home admissions were negatively correlated with COVID-19 IR, while liquor stores remained positive in 2022.

#### 4.2.2 Children

The factor of religion increased the COVID-19 infection at the early stage of the pandemic (*r* = 0.25), but decreased it in 2022 (*r* = -0.22). Likewise, factor- school and healthcare access was positively associated with the IR in 2020 but turned inverse in 2022. Other factors, such as occupation and language, mobility, policies, liquor stores, asthma, race and chronic diseases, healthcare providers, and nursing homes, also had inconsistent effects over the years.

#### 4.2.3 Adults

As indicated by factors 4, 5, 7, and 8, households with no children and better natural amenities decreased the COVID-19 IR, while more school and healthcare access as well as religious gathering increased the IR in 2020. Nevertheless, factor of religion turned negative in 2022, suggesting the positive outcomes of online gathering, social capital, and community support [68]. Moreover, factors of occupation and language, environmental hazards, healthcare providers, and liquor stores positively impacted COVID-19 exposures in 2021–2022. In contrast, asthma and policies decreased the chances of COVID-19 infections among adults in the later years.

#### 4.2.4 Older adults

Factor 1- comorbidities and social status significantly increased the infection in older adults, while factor -race, political affiliation and chronic diseases, surprisingly, decreased the COVID-19 infection in the first two years. This suggested that White republican older adults with low social status and comorbidities had a higher risk of getting infected. Moreover, living close to schools and hospitals increased the infection rates among older adults in 2020, which might be attributable to the childcare duties of grandparents and high virus transmission in urban areas. In addition, asthma, air quality, natural amenities, and environmental hazards had vital impacts on their IR, emphasizing the importance of the environment, especially for older adults.

### 4.3 Adjustable COVID-19 Potential Exposure Index (ACOVIDPEI) for older adults

To answer the third research question—how to quantify the COVID-19 exposure risk in counties for different age groups, an Adjustable COVID-19 Potential Exposure Index (ACOVIDPEI) is created. An example index for older adults in 2020 has been built, and it is a relative measurement of the COVID-19 risk for each county for adults aged 65 and over. Cardinalities were designated based on all component’s effects on older adults in 2020 (Table 6). For example, comorbidities and low social status (factor 1) were positively correlated with older adults’ IR, therefore, a positive cardinality was assigned (Tables 5 & 6). Meanwhile, factor 3-Race, political affiliation and chronic diseases were negatively correlated with older adults’ IR in 2020, thus a negative cardinality was assigned. The factor scores were placed in an additive model to composite ACOVIDPEI-Older adults for each county.

**Table 6:** Cardinality of components in ACOVIDPEI-Older adults in the early pandemic.

Component	Cardinality for Older Adults in 2020	Name
**1**	**+**	Comorbidities and social status
**2**	**-**	Healthcare providers
**3**	**-**	Race and chronic diseases
**4**	**-**	Households without children
**5**	**+**	School and healthcare access
**6**	**+**	Urbanism
**7**	**-**	Natural amenity
**8**	**+**	Religion
**9**	**+**	Air Quality
**10**	**-**	Nursing homes
**11**	**+**	Environmental hazards
**12**	**-**	Vaccinations
**13**	**-**	Mental health centers

**Fig 3:**
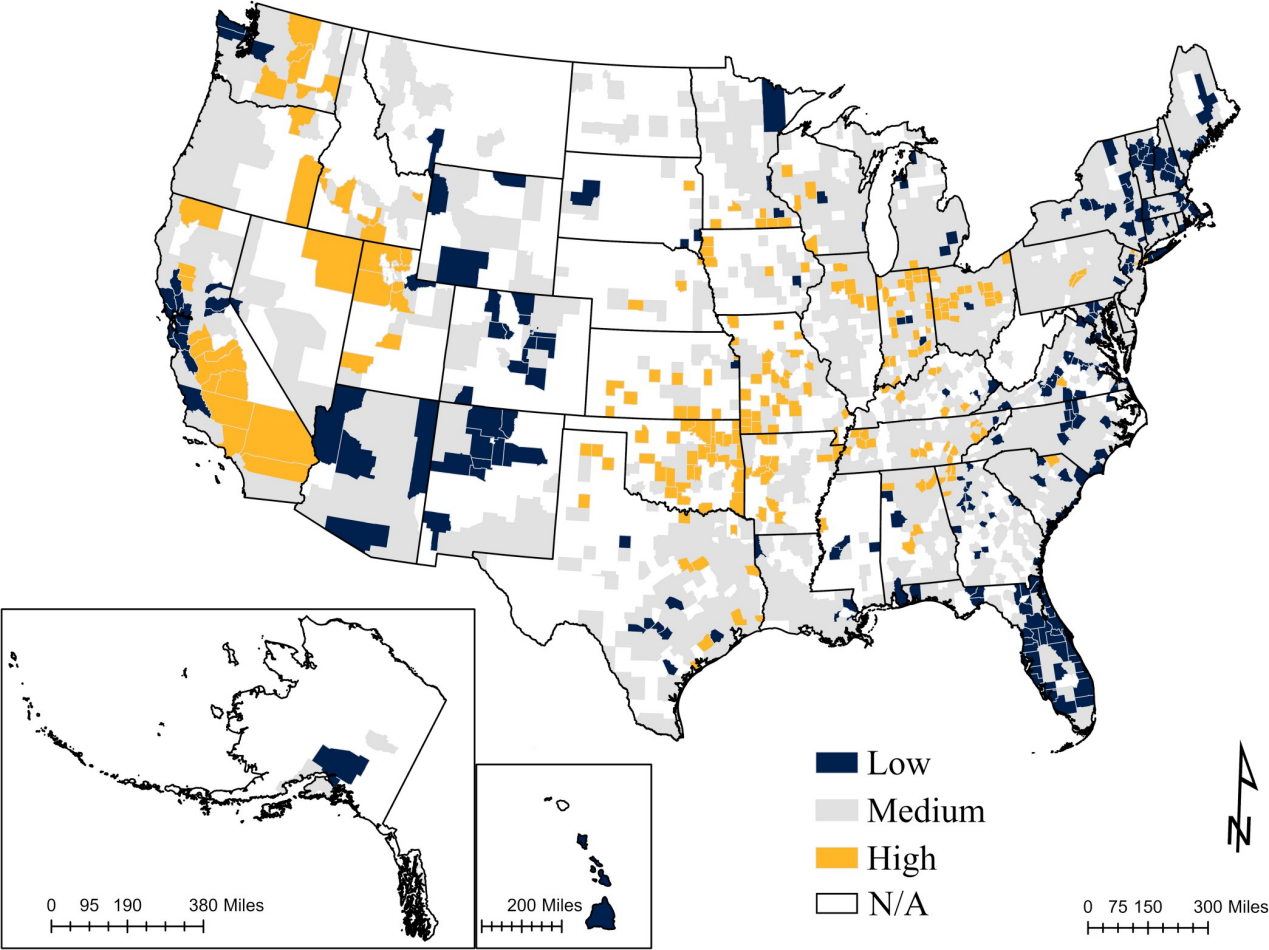
Adjustable COVID-19 exposure (incidence rate in this case) index for older adults in the U.S. counties in 2020. Mapped by standard deviations. Low: <-1 standard deviation; Medium: -1- 1 standard deviation; and High: >1 standard deviation. County and state boundaries are retrieved from the U.S. Census Bureau (https://www.census.gov/geographies/mapping-files/time-series/geo/carto-boundary-file.html). The shapefiles are released under the CC BY 4.0 license.

Counties with ACOVIDPEI-Older Adults scores greater than +1 standard deviation are labeled as the highest COVID-19 exposure risk (Fig 3). They include a geographic mix of high mobility counties and non-Hispanic counties, relatively high access to healthcare, and socially dependent populations. A total of 240 counties (13.7% of the total) were classified in the high-impacted category. Of the high-risk areas, 30.8% (74 out of 240) were metro counties, and 69.2% (166) were nonmetro counties based on RUCC 2013 delineation. The highest potential exposure county in the nation is Holmes County in Ohio, largely based on a high number of households with children, school and healthcare access, and urbanism (Factors 4, 5 & 6). These factors also account for the placement of LaGrange County, Indiana, among the top four highest exposure counties. Both counties have a high percentage of White (98%) and 13% of the population aged 65 and over [69, 70]. Hopewell City, Virginia is ranked second based on factor 11- Environmental hazards, which is heavily decided by the total amount of emissions from the Toxics Release Inventory (TRIs) in the county per square mile. The fifth highest potential exposure county is in Utah, Salt Lake County, and its potential risk is also derived from the Environmental hazards indicators. New York County, New York, is ranked third overall, primarily based on factor 6-Urbanism. As of October 2022, the total number of confirmed cases in New York County was over 6 million and fatalities was over 72,000 [71]. This is not surprising given the counties’ population density and crowded public transportation system. This factor also contributes to the placement of Kings County and Bronx County in New York among the top seven highest exposure counties.

Counties labeled as the least exposure risk (lower than -1 standard deviation from the mean) are clustered in the Northeast, East and West coastal areas, and western states including Wyoming, Colorado, Arizona and New Mexico. Of the low potential exposure counties, 74% of them (179 out of 242) are metro counties and 26% of them are nonmetro counties. Topping the list of least risky counties across the study area are Crisp County, Georgia; St. Johns County, Florida; Hernando County, Florida; St. Tammany Parish, Louisiana; and Perry County, Kentucky. The low potential exposure risk score for Crisp County in Georgia was primarily based on Factor 10-Nursing home. The nursing home admission in Crisp County was 8,890 per 100,000 population, largely due to the high percentage of older adults (18%) and senior care facilities. Even though there were several COVID-19 outbreaks in nursing homes at the beginning of the pandemic, a study confirmed a declining trend in mortality rates from April through November 2020 [72]. They also suggested that the mechanisms driving these trends may include improved clinical management with nursing homes, improved personal protective equipment supply and use, and genetic mutation in the virus. Moreover, the low potential exposure risk scores for St. Johns County, Hernando County, and St. Tammany Parish County were heavily decided by factor 12- Vaccination, which confirmed the significant effects of vaccinations on older adults at the early stage. Additionally, the natural amenity scale provided extra support within the low exposure risk counties. The remaining county, Perry County, has a high density of mental health centers.

To validate the ACOVIDPEI-Older Adults, a Pearson correlation was conducted between the index and older adults’ IR in 2020. The results suggested that ACOVIDPEI-Older Adults was moderately correlated with older adults’ IR (*r* = 0.39, p<0.001). This indicated that the index has better measurement performance than single components.

## 5. Discussion and conclusion

This nationwide COVID-19 individual-level study of over 71 million COVID-19 patients in the U.S. assessed the different distribution of COVID-19 cases by age group from January 2020 through June 2022. Of the 1,748 counties, 961(55%) of them are in metro areas (383 counties with a 1 million population or more; 321 counties with 250,000 to 1 million people; and 257 metro counties have a population fewer than 250,000), while 787(45%) of the total counties are in nonmetro areas. Missing data was due to the suppression of data cells for reliability (case counts below 11) or confidentiality (preventing identifying people) purposes [59]. Another reason might be the lack of healthcare access in rural areas, which also results in insufficient testing capacity and low reporting quality, especially at the early stage of the pandemic.

The majority of COVID-19 infections originated from and were sustained primarily in the adult group [6]. IR in 2021 was higher than in other years, and children were less impacted at the beginning of the pandemic compared with other groups. Geographically, high IR moved from the Midwest, central states, South Carolina, North Carolina, Tennessee, and Arizona to the western and eastern states, then to the west and east coasts. This result corresponds to a previous study that COVID-19 cases were initially high in the Great Plains and Southwestern regions [8]. The governments enacted mitigation measures such as mask mandates, social distancing and stay-at-home orders that can significantly decrease human mobility. During the same time, distinct urban-rural differences showed higher case rates and fatality rates, and fewer government mitigation actions in rural areas [73]. Then in 2021, vaccinations were developed and rapidly deployed among adults. People could not stand “quarantine fatigue” and started to come back to “normal lives” [18]. However, more contagious variants, such as Alpha and Delta, were transmitted across counties, and high IR for all age groups expanded to the entire U.S. Interestingly, the distribution of the IR matched highly with the vaccination rates in 2022 [73]. This might be attributable to residents’ risk perceptions. People are more willing to get vaccinations when the cases and fatalities are increasing in their surroundings [74].

This study proposed the concept of age nonstationarity to indicate the varying effects of health determinants on COVID-19 exposures among age groups. COVID-19 exposure risk is influenced by social, behavioral, environmental, healthcare access, and political contexts. These factors have distinct impacts among different age groups at different time periods during the pandemic. The community-based COVID-19 spatial disparity model considers different levels of geographic level (individual and community), various contextual variables, multiple COVID-19 outcomes, and different geographic contextual units. This model is a synthesis of the genetic factors and determinants of health by different age groups. Breaking apart each of the components of health risk by age group makes it possible to operationalize and measure the influence of each component. This model covers both vertical health inequality (individuals or households) and horizontal inequality (age) for COVID-19 exposure and can be applied to other infectious diseases.

Based on the age-nonstationarity theory and conceptual model, thirteen components have been identified from 62 county-level variables for 1,748 U.S. counties in 2020, and fourteen components were generated in 2021 and 2022. Comorbidities, social status, race, healthcare provider/access, natural amenities, and urbanism were highlighted in all PCA determinants. Some factors had consistent effects, for example, as indicated in the literature, natural amenities were negatively associated with COVID-19 incidences for all age groups across years [65].

Nevertheless, some factors had temporal nonstationarity. For example, factors of race, political affiliation and chronic diseases were negatively correlated with COVID-19 IR in 2020 and 2021, but positively correlated with IR in 2022. This can be supported by the evidence that Black, Hispanic and Asian adults under 65 had a higher vaccination rate than White residents during the first Omicron wave [75]. Moreover, the incomplete data for 2022 after August may change the yearly pattern. Factor of religion also showed a temporal nonstationarity effect- positively correlated with COVID-19 IR in 2020, but negatively associated in 2022. At the beginning of the pandemic, many churches had suggested that social-distancing requirements challenge their “religious freedom” [76]. Around 50 members of Maryville Baptist Church and six other churches in Kentucky attended an in-person Easter 2020 service on this Christian holiday, ignoring the government’s order against mass gatherings and exposing them to high infection risks [77]. However, a survey of 1,500 pastors in April 2020 found most of the churches moved from offline to online-only during the pandemic [78]. Creative religious gathering was later designed such as “parking lot worship”, where people sat in their cars in the church parking lot and listened to the pastor via car radios [79]. It is unknown why religious affiliation was negatively correlated with COVID-19 IR in the middle and later stages of the pandemic. It might be due to the survival effects among members- people were infected and had antibodies from diseases or vaccination; or people passed away and those left were immune and younger [80]. It may also be owing to the religions’ positive impacts on increasing personal resilience, perceptions of community solidarity, and compliance with public health measures [81]. Further studies are needed to explore the role of religion on COVID-19 and other infectious disease exposures.

Certain factors exhibit the age-nonstationarity effects, implying that their effect varies among different age groups. For example, factor of comorbidities and social status was only significant for older adults. This conforms to the literature that comorbidities and lower social status could increase the risk of COVID-19 infection and severe illness consequences [82]. The number of underlying medical conditions increases with age, and older adults with lower socioeconomic status lack adequate healthcare access, nutritious food, and social support.

To further measure the different potential exposure for each age group across counties, the study composited an Adjustable COVID-19 Potential Exposure Index (ACOVIDPEI) and customized it for older adults in 2020 as an example (ACOVIDPEI-Older Adults). The index showed a moderate correlation with the exposures with a better measurement performance than a single set of components. The high potential exposure areas are located in the west, Missouri, South Central states, and East North Central states (Indiana, Ohio, and Wisconsin), while the low potential exposure areas are distributed in coastal California, Wyoming, Colorado, New Mexico, Arizona, and eastern coastal counties. For high-risk counties, 30.8% of them are in metro areas with a high number of older adults with comorbidities or low socio-economic status, less race diversity, and high population density. In terms of low-risk areas, 74% of them are urban areas with better healthcare facilities, good natural amenities, better air quality, and fewer environmental hazards, confirming the importance of healthcare service, environment and green park access on respiratory diseases [83]. The ACOVIDPEI can be adjusted to other age groups or other respiratory diseases by changing the cardinality of the health components. The cardinality can be decided by existing data on the exposures (incidence rates, fatality rates, excess deaths, vaccination rates, etc.) [84–86] or public health expertise decisions at different stages.

This study is not without limitations. First, this patient-level data is not available for all U.S. counties, possibly affecting the results of the COVID-19 incidence pattern. Second, the widespread availability of COVID-19 home testing has caused disruptions in the precision of official reporting positive rates and case numbers. Third, the fatality information is not complete in the dataset, resulting in limited analysis of COVID-19 exposures. Fourth, the use of county-level data in the analysis might be too coarse to ascertain linkages, and a subsequent sub-county level such as census tracts would parse the difference more effectively. In addition, this study employed a year-long interval as its temporal resolution, which may be too coarse for the analysis. In future research, COVID-19 incidence rates across different virus variants waves will be investigated.

This study has unique strengths. First, the study has a large sample size of COVID-19 patients across the U.S. with age information, which provides sufficient statistical power to generate robust results. Second, to the author’s knowledge, this is the first study to develop the concept of age nonstationarity to indicate the varying effects of contextual variables on health among age groups. Furthermore, a community-based COVID-19 spatial disparity model was presented, considering different levels of geographic level (individual and community) and contextual variables. Moreover, a comprehensive list of determinants of health was created. Other studies could use the list as a reference for other infectious diseases analysis. Last but not least, an adjustable COVID-19 Potential Exposure Index was generated. The cardinality for each factor compositing ACOVIDPEI can be adjusted when measuring different community groups and outcomes at different stages of the pandemic. The index can also be applied to assessing other infectious diseases’ impacts. In addition, this index can guide COVID-19 recovery and mitigation policymaking and prepare for other pandemics in different age groups. Usually, a one-size-fits-all strategy is used for mitigation policy actions, but it is inappropriate since it ignores the inherent variability in capabilities not only across counties but also among age groups. To prevent and prepare for other infectious diseases, policymaking needs to consider the differences in population structure, social status, access to healthcare facilities and other public places, the physical environment and scale of interventions as well as its urban-rural character.

## Supporting information

S1 Table Variable names and descriptions. (DOCX)

S2 Table Detailed table for dimensions of COVID-19 related determinants of health in 2020. (DOCX)

S3 Table Detailed table for dimensions of COVID-19 related determinants of health in 2021. (DOCX)

S4 Table Detailed table for dimensions of COVID-19 related determinants of health in 2022 as of June. (DOCX)
