# Supplementary material for: Age-based spatial disparities of COVID-19 incidence rates in the United States counties
Source: PLoS One. 2023 Jun 8;18(6):e0286881. doi: 10.1371/journal.pone.0286881 (PMC10249835; doi:10.1371/journal.pone.0286881)
Supplement: S1 Table — (DOCX) [file pone.0286881.s001.docx]

**S1 Table. Variable names and descriptions.**

| Category | Variable | Type | Description | Data Source | Time frame | Rational Reference |
| --- | --- | --- | --- | --- | --- | --- |
| Political affiliation and intervention | COVID-19 All Policies | Time series | State and county policies (emergency declaration, mask mandates, day care and business closure, stay-at-home) in each year | National Association of Counties; Raifman et al.; U.S. DHHS & U of Chicago; OPENICPSR | 2020-2022 | Jackson et al. 2021; Bendavid et al. 2021; Van Dyke et al. 2020 |
|  | Democratic voters | Static | MIT Election Data and Science, 2021 | MIT Election Data and Science, 2021 | 2020 | Leonhardt 2021 |
| Social economic | Below poverty | Static | Poverty rates | Census - ACS | 2016-2020 | McPhearson et al. 2020 |
|  | Unemployment | Static | Percentage unemployed | U.S. Bureau of Labor Statistics | 2016-2020 | McPhearson et al. 2020 |
|  | Median income | Static | Median household income in last 12 months | Census - ACS | 2016-2020 | McPhearson et al. 2021 |
|  | Income Inequality | Static | GINI Index - Income inequality range (0-1) | County Health Rankings | 2016-2020 | Elgar et al. 2020 |
|  | Population growth | Static | Percentage population change (2010-2020) | Census 2020 | 2016-2020 | Buck 2014; Shiels et al. 2021 |
|  | Health insurance | Static | Percentage of population without health insurance | SAHIE | 2016-2020 | McPhearson et al. 2020 |
|  | Renter | Static | Tenure - percentage of renters | Census - ACS | 2016-2020 | Buck 2014; McPhearson et al. 2021 |
|  | Married population | Static | Percentage of pop (>15) now married | Census - ACS | 2016-2020 | Drefahl et al. 2020 |
|  | Gender | Static | Percentage of female | Census - ACS | 2016-2020 | Cutter 1996; Drefahl et al. 2020 |
|  | Race - Non-white | Static | Percentage of population not classified as white | Census - ACS | 2016-2020 | McPhearson et al. 2020 |
|  | Language/ ability to speak English | Static | Percentage of population not speaking English well for the population 5 years old and over | Census - ACS | 2016-2020 | McPhearson et al. 2020 |
|  | Female Headed Households | Static | Percent Female Headed Households | Census - ACS | 2016-2020 | Cutter 1996 |
|  | Households with children | Static | Percentage of households with own children under 18 years | Census - ACS | 2016-2020 | Fosco et al. 2021 |
|  | Educational attainment - college | Static | Percentage of population with less than 12th Grade Education for the population 25 years and over | Census - ACS | 2016-2020 | Drefahl et al. 2020 |
|  | Healthcare related occupation | Static | Percentage working as healthcare practitioners and technical occupations and health care support occupations for 16 years old and over | Economic Census | 2016-2020 | Hawkins et al. 2021 |
|  | Population density | Static | Number of people per square mile | Census - ACS | 2016-2020 | Bhadra et al. 2021 |
|  | Population with disability | Static | Percentage of population with disability | Statista | 2016-2020 | Morrow 1999 |
|  | Housing Units with No Car | Static | Percent of Housing Units with No Car | Census - ACS | 2016-2020 | Cutter 2003 |
| Behavior, perception, and comorbidity | Hypertension | Static | Age-adjusted prevalence* of hypertension among adults aged >=18 years | CDC PLACES | 2018-2021 | Wan et al. 2020; Ejaz et al. 2020 |
|  | Cardiovascular diseases (CVD) | Static | Age-adjusted prevalence of coronary heart disease among adults aged >=18 years | CDC PLACES | 2018-2021 | Yang et al. 2020 |
|  | Stroke | Static | Age-adjusted prevalence of stroke among adults aged >=18 years | CDC PLACES | 2018-2021 | Uhlen et al. 2015 |
|  | Mental Health | Static | Age-adjusted prevalence of mental health not good for >=14 days among adults aged >=18 years | CDC PLACES | 2018-2021 | Chen et al 2020; Tausch et al. 2022 |
|  | Asthma | Static | Age-adjusted prevalence of asthma among adults aged >=18 years | CDC PLACES | 2018-2021 | Dong et al. 2020 |
|  | HIV | Static | HIV prevalence (per 100000) among people age 13 years and older in 2020. Missing data is replaced by state average. | CDC HIV Statistics Center & Alaska HIV Surveillance Report | 2019 & 2020 | Yasri et al. 2020 |
|  | Diabetes | Static | Age-adjusted prevalence of Diabetes among adults aged >=18 years | CDC PLACES | 2018-2021 | Maddaloni et al. 2020 |
|  | Depression | Static | Age-adjusted prevalence of depression among adults aged >=18 years | CDC PLACES | 2018-2021 | Ustun 2021 |
|  | Religious affiliation | Static | County level congregation membership per 100,000 population | US Religious Census | 2010 | Dein et al. 2020 |
|  | Alcohol | Static | Percentage of population (>=18) who consume > 5 (male) or 4 (female) alcoholic beverages at a time | BRFSS – Cnty Health Rank | 2022 | Zhong et al. 2021 |
|  | Physical inactivity | Static | Percentage of adults that report no leisure-time physical activity | BRFSS – Cnty Health Rank | 2022 | Jiménez-Pavón et al. 2020 |
|  | Obesity | Static | Percentage of population (>=18) with BMI > =30 | BRFSS – Cnty Health Rank | 2022 | Ejaz et al. 2020 |
|  | Low Birth Weight | Static | Percentage of births with low birthweight (<2500g) | BRFSS – Cnty Health Rank | 2022 | Crispi et al. 2021 |
|  | Social associations | Static | Associations per 10,000 population | BRFSS – Cnty Health Rank | 2022 | El-Zoghby et al. 2020 |
|  | Cancer (excluding skin cancer) | Static | Age-adjusted prevalence of cancer (excluding skin cancer) among adults aged >=18 years, 2019 | CDC PLACES | 2018-2021 | Juanjuan et al. 2020 |
|  | COVID-19 vaccination rates | Time series | Percent of people who are fully vaccinated (have second dose of a two-dose vaccine or one dose of a single-dose vaccine) based on the jurisdiction and county where recipient lives in 2020, 2021, and 2022 (as of June 30) | CDC-NPCR | 2020-2022 | Lo Vecchio et al. 2019 |
| Environ-mental | Access to Parks | Static | Percent of people living within a half mile of a park | CDC EPH tracking | 2015 | Slater et al. 2020 |
|  | Recreation facilities | Static | Number of arts, entertainment, and recreational facilities per 100000 | Census Economic Survey 2020 | 2020 | Slater et al. 2020 |
|  | Food environment index | Static | Indicator of access to healthy foods - 0 is worst, 10 is best | County Health Rankings | 2022 |  |
|  | Workplace mobility change | Time series | Movement trends change of workplace (average daily change from baseline between 2/15/2020-6/30/2022) for each year | COVID-19 community mobility reports | 2020-2022 | Jacobsen & Jacobsen 2020 |
|  | School | Static | Number of public and private schools per 100000 population | National Center for Education Statistics | 2022 | Kampe et al. 2020 |
|  | Liquor Store Density | Static | Number of liquor stores per 100000 population. A liquor store is defined as a business that primarily sells packaged alcoholic beverages, such as beer, wine, and spirits. | CDC Economic Survey | 2020 | Zhong et al. 2021 |
|  | Grocery and pharmacy mobility change | Time series | Movement trends change of groceries and pharmacies (average daily change from baseline between 2/15/2020-6/30/2022) for each year | COVID-19 community mobility reports | 2020-2022 | Jacobsen & Jacobsen 2020 |
|  | Natural Amenities Scale | Static | Index for livability of area based on climate factors | USDA-ERS | 2019 | Buck 2014; Slater et al. 2020 |
|  | Environmental hazards | Static | Total amount of emissions from Toxics Release Inventory (TRIs) in county per square mile | EPA-Toxics Release Inventory (TRI) Program | 2020 | Quigley et al. 2020 |
|  | Rural population | Static | Percent of households living in rural areas | Census - DEC Summary File | 2010 | Jackson et al. 2021 |
|  | Particulate Matter Days | Static | Average daily amount of fine particulate matter (PM2.5) in micrograms per cubic meter | County Health Rankings | 2022 | Contini & Costabile 2020 |
|  | Ozone Days | Static | Number of days with maximum 8-hour average ozone concentration over the National Ambient Air Quality Standard (0.08ppm(v) or greater) (monitor and modeled data) | CDC EPH tracking | 2018 | Contini & Costabile 2020 |
|  | Violent Crime | Static | Violent crimes per 100,000 population | BRFSS – Cnty Health Rank | 2020-2021 | Som et al. 2020 |
| Healthcare provider and access | Number of doctors | Static | Number of Primary Care Physicians (patient care and hospital resident) per 100,000 population, exclude 75+ in 2019 | HRSA Area Health Resources Files | 2021 | Ahmed et al. 2020 |
|  | Number of internal MDs | Static | Number of MD per 100,000 population in 2019 | HRSA Area Health Resources Files | 2021 | Ahmed et al. 2020 |
|  | Hospitals | Static | Number of hospitals per 100,000 population in 2019 | HRSA Area Health Resources Files | 2021 | Noronha et al. 2020 |
|  | Pharmacies | Static | Number of pharmacies per 100,000 population | HIFLD | 2021 | Bahloli & Dewey 2021 |
|  | Nursing homes admissions | Static | Nursing Home Admissions (total hospitals) per 100,000 population in 2019 | HRSA Area Health Resources Files | 2021 | Bahloli & Dewey 2021 |
|  | Primary care providers | Static | Number of Pediatrician per 100,000 population | HRSA Area Health Resources Files | 2020-2021 | Greenhalgh et al. 2020 |
|  | Emergency departments visits | Static | Number of emergency departments visits per 100,000 population in 2019 | HRSA Area Health Resources Files | 2021 | Parri et al. 2020 |
|  | ICU beds | Static | Number of hospital beds per 100,000 population in 2019 | HRSA Area Health Resources Files | 2020-2021 | Noronha et al. 2020 |
|  | Mobile van sites | Static | Number of mobile van sites per 100,000 population | Mobile Health Map | 2022 | Wenzel & Fishman 2021 |
|  | Mental health centers | Static | Number of mental health centers per 100,000 population in 2020 | HRSA Area Health Resources Files | 2020-2021 | Moreno et al. 2020 |
|  | Telehealth service provided by hospitals | Static | Number of Telehealth services provided by hospitals with per 100,000 in 2019 | HRSA Area Health Resources Files | 2020-2021 | Monaghesh & Hajizadeh 2020 |
|  | FEMA Federal Support | Time series | FEMA obligation amount per hospital in the state (only in 2020) | FEMA | 2020 | Ahmed et al. 2020 |
|  | Medically Underserved Areas/population | Static | Percentage of MUA areas of the county | Health Resources & Services Administration (HRSA) | 2021 | Woodall et al. 2020 |

*The missing values for counties were replaced by the state average or national average if the state value is missing.
