# Supplementary material for: Age-based spatial disparities of COVID-19 incidence rates in the United States counties
Source: PLoS One. 2023 Jun 8;18(6):e0286881. doi: 10.1371/journal.pone.0286881 (PMC10249835; doi:10.1371/journal.pone.0286881)
Supplement: S2 Table — (DOCX) [file pone.0286881.s002.docx]

**S2 Table. Detailed table for dimensions of COVID-19 related determinants of health in 2020.**

| Variables | Components Loading | | | | | | | | | | | | |
| --- | --- | --- | --- | --- | --- | --- | --- | --- | --- | --- | --- | --- | --- |
|  | **1** | **2** | **3** | **4** | **5** | **6** | **7** | **8** | **9** | **10** | **11** | **12** | **13** |
| % Variance Explained | 21.889 | 8.293 | 8.140 | 4.956 | 4.285 | 3.892 | 3.416 | 3.296 | 2.972 | 2.102 | 2.063 | 1.979 | 1.921 |
| COVID-19 All Policies | -0.170 | 0.216 | 0.150 | 0.186 | -0.146 | 0.058 | 0.021 | -0.434 | 0.209 | -0.142 | 0.010 | -0.094 | 0.155 |
| Democratic voters | -0.311 | 0.451 | 0.588 | 0.130 | -0.151 | 0.186 | 0.058 | -0.250 | -0.017 | -0.035 | 0.077 | -0.156 | -0.089 |
| Hypertension | 0.852 | -0.054 | 0.097 | 0.012 | -0.181 | -0.027 | -0.196 | 0.188 | -0.070 | 0.038 | 0.042 | 0.005 | 0.113 |
| Cardiovascular diseases (CVD) | 0.933 | -0.111 | -0.171 | 0.040 | 0.080 | 0.045 | -0.067 | 0.040 | -0.045 | 0.011 | -0.023 | 0.003 | -0.006 |
| Stroke | 0.935 | -0.057 | 0.165 | 0.070 | -0.022 | 0.040 | -0.054 | 0.059 | -0.068 | 0.018 | 0.031 | -0.001 | 0.002 |
| Mental Health | 0.865 | -0.110 | -0.303 | 0.166 | -0.074 | 0.035 | -0.062 | -0.039 | -0.038 | 0.016 | 0.056 | -0.025 | 0.056 |
| Asthma | 0.563 | 0.021 | -0.323 | 0.377 | -0.009 | 0.146 | -0.164 | -0.277 | -0.074 | 0.042 | 0.200 | -0.241 | -0.054 |
| HIV | 0.220 | 0.219 | 0.638 | 0.024 | -0.235 | 0.424 | 0.046 | 0.037 | -0.066 | 0.018 | 0.075 | 0.059 | 0.074 |
| Diabetes | 0.847 | -0.042 | 0.371 | -0.189 | -0.044 | 0.021 | -0.062 | 0.107 | 0.090 | 0.018 | -0.045 | 0.027 | 0.012 |
| Depression | 0.539 | 0.007 | -0.668 | 0.158 | -0.110 | -0.018 | -0.079 | -0.052 | -0.075 | -0.083 | 0.116 | -0.015 | 0.040 |
| Religious affiliation | 0.088 | 0.140 | 0.095 | -0.071 | 0.126 | -0.018 | -0.151 | 0.700 | 0.131 | 0.006 | 0.071 | 0.000 | 0.098 |
| Alcohol | -0.638 | -0.086 | 0.074 | 0.388 | 0.182 | 0.024 | -0.053 | -0.044 | -0.089 | -0.050 | -0.131 | 0.163 | -0.140 |
| Physical inactivity | 0.858 | -0.160 | 0.027 | -0.102 | 0.043 | 0.061 | -0.179 | 0.197 | 0.021 | 0.054 | -0.036 | 0.025 | 0.018 |
| Obesity | 0.704 | -0.199 | 0.029 | 0.018 | 0.145 | -0.139 | -0.443 | 0.146 | 0.022 | 0.031 | 0.033 | 0.024 | -0.045 |
| Low Birth Weight | 0.622 | 0.088 | 0.391 | 0.180 | -0.289 | -0.041 | 0.034 | 0.159 | -0.131 | 0.053 | 0.162 | 0.001 | 0.133 |
| Social associations | -0.060 | 0.078 | -0.136 | 0.456 | 0.283 | -0.025 | -0.225 | 0.478 | -0.178 | -0.049 | 0.049 | -0.154 | -0.098 |
| Cancer | -0.021 | -0.037 | -0.748 | 0.365 | -0.085 | -0.055 | -0.204 | -0.042 | -0.279 | 0.013 | 0.092 | -0.054 | 0.013 |
| COVID vaccination rates | -0.026 | -0.006 | -0.009 | 0.077 | -0.094 | 0.061 | 0.065 | 0.046 | 0.012 | 0.092 | 0.070 | 0.577 | 0.035 |
| Access to Parks | -0.404 | 0.357 | 0.189 | -0.035 | 0.134 | 0.186 | 0.342 | -0.220 | 0.124 | -0.089 | 0.250 | -0.141 | 0.028 |
| Recreation facilities | -0.523 | 0.223 | -0.027 | 0.149 | 0.151 | 0.133 | 0.391 | 0.136 | -0.234 | -0.030 | 0.063 | -0.133 | -0.092 |
| Food environment index | -0.808 | -0.063 | -0.029 | 0.004 | -0.154 | 0.122 | -0.139 | 0.021 | -0.012 | 0.050 | -0.077 | -0.069 | 0.052 |
| Workplace mobility change | 0.433 | -0.437 | -0.330 | 0.174 | 0.341 | -0.179 | -0.036 | 0.272 | -0.139 | 0.054 | 0.031 | 0.078 | -0.087 |
| School | 0.004 | -0.167 | -0.144 | 0.086 | 0.705 | 0.033 | -0.088 | 0.056 | 0.013 | -0.082 | -0.093 | -0.015 | -0.101 |
| Liquor Store Density | -0.242 | 0.079 | 0.105 | 0.220 | -0.053 | 0.126 | 0.130 | 0.110 | -0.076 | 0.143 | -0.020 | -0.542 | 0.105 |
| Grocery and pharmacy mobility change | 0.158 | -0.326 | -0.401 | 0.150 | 0.192 | -0.061 | -0.002 | 0.128 | -0.051 | 0.092 | 0.198 | 0.042 | -0.117 |
| Natural Amenities Scale | 0.009 | 0.055 | 0.138 | -0.163 | -0.033 | -0.060 | 0.807 | -0.196 | 0.199 | -0.014 | 0.015 | 0.010 | 0.048 |
| Environmental hazards | 0.002 | -0.049 | 0.086 | -0.008 | -0.044 | 0.048 | 0.008 | 0.034 | 0.058 | 0.029 | 0.694 | 0.105 | 0.050 |
| Rural population | 0.305 | -0.504 | -0.411 | 0.225 | 0.155 | -0.033 | 0.048 | 0.027 | -0.221 | 0.020 | -0.255 | 0.057 | -0.034 |
| Particulate matter days | 0.134 | 0.034 | 0.040 | -0.088 | -0.101 | 0.019 | -0.074 | 0.076 | 0.758 | -0.111 | -0.007 | 0.106 | -0.045 |
| Ozone days | -0.135 | 0.006 | 0.138 | -0.011 | -0.028 | 0.025 | 0.234 | -0.059 | 0.715 | 0.042 | 0.095 | -0.070 | -0.038 |
| Violent crime | 0.363 | 0.229 | 0.501 | 0.068 | -0.102 | 0.082 | 0.081 | 0.122 | 0.069 | -0.073 | 0.232 | 0.008 | -0.101 |
| Number of primary care physicians | -0.305 | 0.853 | 0.060 | 0.114 | 0.000 | 0.055 | 0.058 | 0.065 | -0.025 | 0.031 | -0.050 | 0.014 | 0.029 |
| Number of internal MDs | -0.249 | 0.842 | 0.131 | 0.065 | -0.087 | 0.127 | 0.042 | 0.049 | -0.010 | 0.009 | -0.087 | 0.054 | 0.032 |
| Hospitals | 0.198 | 0.025 | -0.028 | 0.181 | 0.607 | -0.088 | 0.001 | 0.277 | -0.090 | 0.207 | 0.065 | 0.046 | 0.124 |
| Pharmacies | 0.355 | 0.182 | -0.162 | 0.232 | 0.016 | 0.177 | 0.099 | 0.351 | -0.105 | 0.092 | -0.007 | -0.084 | 0.372 |
| Nursing homes admissions | 0.030 | 0.053 | -0.018 | -0.008 | 0.042 | 0.032 | -0.030 | -0.016 | -0.070 | 0.752 | 0.018 | 0.050 | -0.149 |
| Pediatrics | -0.228 | 0.798 | 0.184 | -0.015 | -0.177 | 0.123 | 0.022 | 0.024 | 0.018 | 0.023 | -0.080 | 0.020 | 0.053 |
| Emergency departments visits | 0.222 | 0.449 | -0.003 | 0.073 | 0.264 | -0.030 | -0.029 | 0.047 | -0.040 | 0.295 | 0.172 | -0.092 | 0.116 |
| ICU beds | 0.118 | 0.504 | 0.146 | 0.137 | 0.242 | 0.000 | -0.078 | 0.168 | -0.051 | 0.501 | 0.123 | 0.019 | 0.164 |
| Mobile van sites | 0.013 | 0.160 | 0.150 | 0.017 | 0.155 | 0.078 | -0.037 | -0.152 | -0.109 | -0.231 | 0.114 | 0.224 | 0.011 |
| Mental health centers | 0.066 | 0.056 | 0.004 | -0.020 | 0.108 | 0.004 | 0.010 | -0.009 | -0.043 | -0.115 | 0.046 | 0.007 | 0.748 |
| Telehealth service provided by hospitals | -0.063 | 0.125 | -0.132 | 0.032 | 0.469 | -0.028 | 0.045 | 0.114 | -0.111 | 0.067 | 0.069 | -0.130 | 0.156 |
| FEMA Federal Support | -0.211 | -0.100 | 0.142 | 0.179 | -0.100 | 0.466 | 0.121 | -0.164 | 0.177 | 0.316 | -0.181 | -0.200 | 0.030 |
| Medically Underserved Areas/population | 0.536 | -0.214 | 0.059 | 0.017 | -0.148 | -0.086 | 0.354 | 0.076 | -0.149 | -0.046 | -0.109 | 0.168 | 0.053 |
| Below poverty | 0.863 | 0.149 | 0.161 | 0.075 | 0.150 | 0.087 | 0.099 | -0.046 | -0.004 | -0.005 | 0.023 | -0.020 | -0.105 |
| Unemployment | 0.801 | -0.180 | 0.079 | 0.111 | 0.012 | 0.037 | 0.240 | -0.127 | 0.096 | 0.032 | -0.170 | 0.111 | 0.141 |
| Median income | -0.813 | 0.058 | 0.078 | -0.235 | -0.292 | 0.029 | -0.042 | -0.066 | 0.098 | 0.038 | -0.066 | -0.022 | 0.152 |
| Income Inequality | 0.400 | 0.484 | 0.211 | 0.170 | -0.092 | 0.182 | 0.351 | 0.096 | -0.006 | -0.043 | -0.104 | 0.020 | -0.089 |
| Population growth | -0.336 | 0.153 | 0.057 | -0.584 | -0.307 | -0.163 | 0.193 | -0.040 | -0.051 | -0.038 | 0.019 | 0.236 | 0.019 |
| Health insurance | 0.493 | -0.083 | 0.177 | -0.493 | 0.119 | -0.031 | 0.224 | 0.203 | -0.087 | -0.043 | -0.111 | 0.105 | -0.122 |
| Renter | 0.191 | 0.550 | 0.422 | -0.168 | 0.118 | 0.304 | 0.037 | -0.058 | 0.057 | -0.037 | 0.224 | -0.140 | -0.115 |
| Married population | -0.392 | -0.398 | -0.541 | -0.181 | -0.108 | -0.178 | 0.017 | 0.211 | 0.066 | 0.005 | -0.108 | 0.123 | 0.124 |
| Gender | 0.091 | 0.352 | -0.054 | 0.141 | -0.516 | 0.027 | -0.059 | 0.276 | 0.000 | 0.009 | 0.233 | -0.069 | -0.113 |
| Race - Non-white | 0.300 | 0.202 | 0.758 | -0.105 | -0.238 | 0.115 | 0.009 | 0.015 | 0.031 | 0.014 | 0.110 | -0.026 | 0.022 |
| Language/ability to speak English | 0.158 | 0.006 | 0.180 | -0.302 | -0.055 | 0.167 | 0.070 | 0.306 | 0.112 | -0.131 | -0.129 | -0.147 | -0.044 |
| Female Headed Households | 0.647 | 0.082 | 0.556 | -0.027 | -0.180 | 0.090 | -0.111 | 0.013 | 0.106 | 0.092 | 0.093 | -0.037 | 0.069 |
| Households with children | -0.158 | -0.079 | 0.097 | -0.671 | -0.056 | -0.101 | -0.340 | 0.037 | 0.326 | 0.070 | -0.022 | 0.039 | 0.159 |
| Educational attainment - college | 0.710 | -0.190 | 0.184 | -0.275 | 0.134 | 0.165 | 0.085 | 0.084 | 0.198 | -0.011 | -0.207 | 0.036 | 0.023 |
| Healthcare related occupation | 0.177 | 0.272 | -0.091 | 0.567 | 0.059 | -0.033 | -0.111 | -0.046 | 0.095 | 0.090 | -0.123 | 0.217 | 0.163 |
| Population density | -0.109 | 0.211 | 0.148 | -0.088 | -0.090 | 0.848 | 0.013 | 0.038 | 0.006 | -0.050 | 0.075 | 0.096 | 0.048 |
| Population with disability | 0.707 | -0.177 | -0.283 | 0.328 | 0.075 | -0.023 | 0.173 | -0.035 | -0.053 | 0.011 | 0.009 | 0.081 | 0.093 |
| Housing Units with No Car | 0.225 | 0.253 | 0.210 | 0.148 | 0.098 | 0.831 | -0.073 | -0.017 | -0.009 | 0.016 | 0.053 | -0.025 | -0.037 |

1. Extraction Method: Principal Component Analysis. Rotation Method: Varimax with Kaiser Normalization.
2. Factor 1- Comorbidities and Social Status, Factor 2- Healthcare Providers, Factor 3- Race and Chronic Disease, Factor 4- Households without Children, Factor 5- School and Healthcare Access, Factor 6- Urbanism, Factor 7- Natural Amenity, Factor 8- Religion, Factor 9- Air Quality, Factor 10- Nursing Homes, Factor 11- Environmental Hazards, Factor 12- Vaccinations, and Factor 13- Mental Health Centers.
