# Supplementary material for: Age-based spatial disparities of COVID-19 incidence rates in the United States counties
Source: PLoS One. 2023 Jun 8;18(6):e0286881. doi: 10.1371/journal.pone.0286881 (PMC10249835; doi:10.1371/journal.pone.0286881)
Supplement: S3 Table — (DOCX) [file pone.0286881.s003.docx]

**S3 Table. Detailed table for dimensions of COVID-19 related determinants of health in 2021.**

| Variables | Components Loading | | | | | | | | | | | | | |
| --- | --- | --- | --- | --- | --- | --- | --- | --- | --- | --- | --- | --- | --- | --- |
|  | **1** | **2** | **3** | **4** | **5** | **6** | **7** | **8** | **9** | **10** | **11** | **12** | **13** | **14** |
| % Variance Explained | 22.480 | 9.161 | 6.810 | 4.696 | 3.901 | 3.704 | 3.571 | 3.506 | 2.749 | 2.600 | 2.566 | 2.040 | 1.984 | 1.885 |
| COVID-19 All Policies | -0.078 | 0.167 | -0.059 | -0.169 | 0.013 | 0.144 | -0.103 | 0.090 | 0.021 | 0.644 | 0.199 | 0.148 | -0.160 | 0.148 |
| Democratic voters | -0.390 | 0.681 | 0.299 | -0.145 | 0.119 | 0.147 | -0.119 | 0.142 | -0.048 | 0.142 | -0.099 | -0.047 | -0.009 | -0.111 |
| Hypertension | 0.871 | 0.098 | 0.020 | -0.107 | -0.231 | -0.052 | -0.019 | -0.021 | -0.054 | 0.065 | 0.056 | 0.025 | 0.107 | 0.073 |
| Cardiovascular diseases (CVD) | 0.936 | -0.079 | -0.071 | 0.095 | -0.067 | 0.023 | 0.081 | 0.047 | -0.031 | -0.037 | -0.111 | 0.009 | -0.049 | 0.000 |
| Stroke | 0.929 | 0.216 | -0.042 | 0.005 | -0.062 | 0.020 | 0.036 | 0.045 | -0.058 | 0.038 | 0.011 | 0.028 | 0.020 | -0.011 |
| Mental Health | 0.877 | -0.200 | -0.042 | -0.032 | -0.064 | 0.131 | 0.139 | 0.046 | -0.037 | 0.044 | -0.203 | 0.006 | 0.052 | 0.030 |
| Asthma | 0.531 | -0.093 | -0.007 | 0.037 | -0.117 | 0.349 | 0.160 | 0.125 | -0.097 | 0.132 | -0.549 | -0.008 | 0.069 | -0.087 |
| HIV | 0.185 | 0.623 | 0.161 | -0.223 | 0.037 | -0.039 | -0.077 | 0.434 | -0.043 | 0.138 | 0.199 | 0.072 | 0.090 | 0.067 |
| Diabetes | 0.839 | 0.352 | -0.044 | -0.035 | -0.040 | -0.177 | -0.166 | 0.006 | 0.082 | -0.047 | 0.136 | 0.023 | -0.010 | -0.021 |
| Depression | 0.552 | -0.506 | 0.109 | -0.054 | -0.085 | 0.146 | 0.219 | 0.020 | -0.069 | -0.100 | -0.362 | -0.093 | 0.100 | 0.020 |
| Religious affiliation | 0.126 | -0.007 | 0.280 | 0.331 | -0.322 | -0.312 | -0.019 | 0.000 | 0.170 | 0.025 | 0.357 | -0.077 | 0.263 | 0.001 |
| Alcohol | -0.636 | 0.024 | -0.119 | 0.114 | -0.110 | 0.316 | 0.267 | 0.050 | -0.057 | 0.003 | 0.244 | -0.002 | -0.143 | -0.066 |
| Physical inactivity | 0.872 | 0.031 | -0.113 | 0.070 | -0.196 | -0.130 | -0.046 | 0.053 | 0.044 | -0.037 | 0.037 | 0.036 | -0.016 | 0.016 |
| Obesity | 0.703 | 0.056 | -0.199 | 0.147 | -0.444 | -0.013 | 0.022 | -0.129 | 0.047 | -0.148 | -0.018 | 0.047 | 0.005 | -0.036 |
| Low Birth Weight | 0.627 | 0.379 | 0.154 | -0.174 | -0.030 | 0.052 | 0.044 | -0.028 | -0.120 | 0.170 | 0.154 | 0.005 | 0.259 | 0.061 |
| Social associations | -0.044 | -0.118 | 0.152 | 0.449 | -0.429 | 0.066 | 0.371 | 0.011 | -0.096 | 0.271 | 0.120 | -0.064 | 0.048 | -0.093 |
| Cancer | 0.007 | -0.615 | 0.064 | -0.030 | -0.262 | 0.279 | 0.347 | -0.030 | -0.248 | 0.071 | -0.340 | 0.002 | 0.050 | 0.046 |
| COVID vaccination rates | -0.563 | 0.251 | 0.223 | 0.004 | 0.201 | 0.248 | -0.105 | 0.062 | 0.018 | 0.033 | -0.100 | -0.193 | -0.062 | -0.101 |
| Access to Parks | -0.476 | 0.327 | 0.208 | 0.106 | 0.390 | -0.037 | 0.010 | 0.160 | 0.111 | 0.004 | -0.243 | -0.072 | 0.078 | 0.068 |
| Recreation facilities | -0.538 | 0.008 | 0.237 | 0.230 | 0.280 | -0.070 | 0.272 | 0.140 | -0.201 | 0.143 | 0.032 | -0.064 | 0.040 | -0.104 |
| Food environment index | -0.776 | -0.143 | -0.032 | -0.145 | -0.163 | -0.004 | -0.112 | 0.103 | -0.026 | 0.183 | 0.073 | 0.030 | -0.013 | 0.014 |
| Workplace mobility change | 0.327 | -0.315 | -0.293 | 0.268 | -0.115 | 0.001 | 0.470 | -0.151 | -0.086 | -0.079 | 0.016 | 0.063 | -0.038 | -0.013 |
| School | 0.006 | -0.156 | -0.235 | 0.692 | -0.055 | 0.122 | -0.079 | 0.075 | -0.014 | -0.121 | -0.003 | -0.078 | -0.127 | -0.162 |
| Liquor Store Density | -0.239 | 0.113 | 0.103 | 0.126 | 0.039 | -0.101 | 0.057 | 0.028 | -0.063 | 0.631 | -0.234 | -0.045 | -0.025 | -0.017 |
| Grocery and pharmacy mobility change | 0.033 | -0.149 | -0.123 | -0.042 | -0.058 | 0.003 | 0.631 | -0.097 | -0.014 | -0.111 | -0.075 | 0.175 | -0.040 | 0.050 |
| Natural Amenities Scale | -0.008 | 0.107 | 0.042 | -0.067 | 0.848 | -0.097 | -0.027 | -0.061 | 0.162 | 0.047 | 0.062 | -0.037 | 0.021 | 0.017 |
| Environmental hazards | -0.002 | 0.125 | -0.067 | 0.035 | 0.024 | 0.075 | -0.064 | 0.063 | 0.021 | -0.085 | -0.013 | 0.014 | 0.766 | -0.002 |
| Rural population | 0.371 | -0.510 | -0.377 | 0.148 | 0.010 | 0.203 | 0.167 | 0.018 | -0.227 | 0.061 | 0.120 | -0.010 | -0.160 | -0.101 |
| Particulate matter days | 0.126 | 0.018 | 0.014 | -0.157 | -0.050 | -0.051 | -0.072 | 0.047 | 0.790 | -0.065 | 0.076 | -0.010 | -0.040 | 0.013 |
| Ozone days | -0.151 | 0.134 | -0.029 | -0.043 | 0.263 | 0.006 | -0.054 | -0.001 | 0.718 | 0.055 | -0.048 | 0.003 | 0.074 | -0.038 |
| Violent crime | 0.321 | 0.598 | 0.194 | -0.076 | 0.012 | -0.082 | 0.136 | 0.062 | 0.117 | -0.036 | 0.063 | -0.068 | 0.165 | -0.019 |
| Number of primary care physicians | -0.351 | 0.192 | 0.813 | 0.090 | 0.039 | 0.112 | -0.048 | 0.069 | -0.036 | -0.010 | -0.034 | 0.039 | -0.072 | 0.036 |
| Number of internal MDs | -0.293 | 0.236 | 0.804 | -0.010 | 0.038 | 0.090 | -0.106 | 0.148 | -0.027 | -0.032 | 0.011 | 0.036 | -0.080 | 0.025 |
| Hospitals | 0.208 | -0.036 | 0.026 | 0.707 | -0.056 | 0.090 | 0.048 | -0.062 | -0.074 | 0.010 | 0.127 | 0.134 | 0.091 | 0.076 |
| Pharmacies | 0.386 | -0.151 | 0.325 | 0.212 | -0.031 | 0.007 | 0.154 | 0.176 | -0.065 | 0.265 | 0.064 | -0.022 | 0.126 | 0.275 |
| Nursing homes admissions | 0.017 | -0.018 | 0.030 | 0.041 | -0.010 | 0.021 | 0.092 | 0.062 | -0.002 | 0.049 | 0.011 | 0.815 | -0.012 | -0.121 |
| Pediatrics | -0.268 | 0.274 | 0.764 | -0.101 | 0.035 | 0.039 | -0.181 | 0.127 | -0.012 | -0.012 | 0.000 | 0.037 | -0.052 | 0.030 |
| Emergency departments visits | 0.176 | 0.166 | 0.374 | 0.331 | -0.030 | 0.014 | 0.086 | -0.062 | -0.013 | 0.048 | -0.180 | 0.294 | 0.073 | 0.166 |
| ICU beds | 0.079 | 0.233 | 0.462 | 0.344 | -0.105 | 0.075 | 0.037 | -0.003 | -0.006 | 0.064 | 0.008 | 0.491 | 0.090 | 0.189 |
| Mobile van sites | -0.052 | 0.290 | 0.028 | -0.010 | -0.031 | 0.037 | 0.226 | 0.097 | -0.061 | -0.330 | -0.006 | -0.106 | -0.125 | 0.279 |
| Mental health centers | 0.063 | -0.019 | 0.036 | 0.064 | 0.036 | 0.011 | -0.023 | 0.015 | -0.001 | 0.041 | 0.008 | -0.054 | -0.005 | 0.812 |
| Telehealth service provided by hospitals | -0.072 | -0.086 | 0.085 | 0.527 | 0.030 | -0.054 | 0.037 | -0.020 | -0.107 | 0.029 | -0.099 | 0.055 | 0.023 | 0.107 |
| Medically Underserved Areas/population | 0.572 | -0.047 | -0.100 | -0.131 | 0.315 | 0.019 | 0.080 | -0.035 | -0.150 | 0.001 | 0.308 | -0.060 | 0.037 | -0.001 |
| Below poverty | 0.818 | 0.312 | 0.092 | 0.152 | 0.117 | 0.050 | 0.089 | 0.079 | 0.008 | -0.061 | -0.102 | 0.003 | -0.065 | -0.067 |
| Unemployment | 0.814 | 0.052 | -0.138 | -0.005 | 0.286 | 0.197 | -0.031 | 0.044 | 0.062 | -0.002 | 0.103 | -0.018 | -0.080 | 0.070 |
| Median income | -0.788 | -0.066 | 0.067 | -0.288 | 0.012 | -0.099 | -0.336 | 0.008 | 0.042 | 0.074 | 0.076 | 0.011 | 0.057 | 0.060 |
| Income Inequality | 0.365 | 0.302 | 0.508 | -0.007 | 0.286 | 0.057 | 0.140 | 0.204 | 0.014 | 0.053 | 0.124 | -0.058 | -0.063 | -0.093 |
| Population growth | -0.336 | -0.023 | 0.140 | -0.401 | 0.254 | -0.391 | -0.196 | -0.127 | -0.058 | -0.288 | 0.153 | 0.105 | 0.058 | 0.078 |
| Health insurance | 0.497 | 0.088 | -0.074 | 0.081 | 0.222 | -0.480 | -0.130 | 0.002 | -0.081 | -0.189 | 0.252 | 0.053 | -0.081 | -0.112 |
| Renter | 0.093 | 0.643 | 0.353 | 0.090 | 0.069 | -0.212 | -0.039 | 0.261 | 0.095 | -0.043 | -0.235 | 0.056 | -0.001 | 0.038 |
| Married population | -0.298 | -0.746 | -0.205 | -0.076 | -0.026 | -0.134 | -0.058 | -0.137 | 0.044 | -0.044 | 0.185 | -0.038 | 0.080 | 0.013 |
| Gender | 0.087 | 0.008 | 0.486 | -0.341 | -0.185 | -0.054 | 0.160 | 0.031 | 0.034 | 0.175 | -0.019 | -0.029 | 0.292 | -0.154 |
| Race - Non-white | 0.259 | 0.738 | 0.124 | -0.225 | 0.035 | -0.107 | -0.219 | 0.094 | 0.019 | 0.100 | 0.167 | 0.035 | 0.116 | -0.005 |
| Language/ability to speak English | 0.160 | 0.168 | 0.044 | -0.045 | -0.041 | -0.526 | 0.037 | 0.095 | 0.163 | 0.107 | 0.101 | -0.134 | -0.229 | 0.056 |
| Female Headed Households | 0.618 | 0.578 | 0.038 | -0.156 | -0.082 | -0.036 | -0.155 | 0.050 | 0.099 | 0.091 | 0.051 | 0.072 | 0.090 | 0.031 |
| Households with children | -0.141 | -0.021 | -0.123 | -0.116 | -0.209 | -0.414 | -0.573 | -0.141 | 0.261 | -0.237 | -0.002 | 0.092 | 0.036 | 0.103 |
| Educational attainment - college | 0.719 | 0.124 | -0.182 | 0.097 | 0.119 | -0.224 | -0.203 | 0.145 | 0.177 | -0.048 | 0.156 | -0.013 | -0.173 | -0.011 |
| Healthcare related occupation | 0.178 | -0.037 | 0.315 | 0.124 | -0.110 | 0.635 | 0.074 | 0.007 | 0.079 | -0.003 | 0.051 | -0.005 | -0.057 | 0.084 |
| Population density | -0.132 | 0.186 | 0.156 | -0.094 | 0.019 | -0.082 | -0.074 | 0.876 | 0.041 | -0.004 | 0.017 | 0.021 | 0.079 | 0.055 |
| Population with disability | 0.721 | -0.219 | -0.092 | 0.099 | 0.146 | 0.298 | 0.262 | 0.010 | -0.031 | 0.024 | -0.050 | -0.036 | 0.014 | 0.085 |
| Housing Units with No Car | 0.178 | 0.341 | 0.159 | 0.115 | -0.068 | 0.088 | 0.004 | 0.828 | 0.019 | 0.086 | -0.105 | 0.045 | -0.019 | -0.026 |

1. Extraction Method: Principal Component Analysis. Rotation Method: Varimax with Kaiser Normalization.
2. Factor 1- Comorbidities and Social Status, Factor 2- Race and Political Affiliation, Factor 3- Healthcare Providers, Factor 4- School and Healthcare Access, Factor 5- Natural Amenity, Factor 6- Occupation and Language, Factor 7- Mobility, Factor 8- Urbanism, Factor 9- Air Quality, Factor 10- Policies and Liquor Stores, Factor 11- Asthma, Factor 12-Nursing Homes, Factor 13- Environmental Hazards, Factor 14- Mental Health Centers.
