# Supplementary material for: Age-based spatial disparities of COVID-19 incidence rates in the United States counties
Source: PLoS One. 2023 Jun 8;18(6):e0286881. doi: 10.1371/journal.pone.0286881 (PMC10249835; doi:10.1371/journal.pone.0286881)
Supplement: S4 Table — (DOCX) [file pone.0286881.s004.docx]

**S4 Table. Detailed table for dimensions of COVID-19 related determinants of health in 2022 as of June.**

| Variables | Components Loading | | | | | | | | | | | | | |
| --- | --- | --- | --- | --- | --- | --- | --- | --- | --- | --- | --- | --- | --- | --- |
|  | **1** | **2** | **3** | **4** | **5** | **6** | **7** | **8** | **9** | **10** | **11** | **12** | **13** | **14** |
| % Variance Explained | 22.242 | 9.017 | 7.679 | 4.395 | 4.282 | 3.583 | 3.540 | 2.978 | 2.806 | 2.762 | 2.353 | 2.147 | 1.944 | 1.895 |
| COVID-19 All Policies | -0.089 | 0.093 | -0.027 | 0.142 | 0.016 | 0.215 | 0.067 | -0.154 | 0.025 | -0.093 | -0.709 | 0.039 | -0.065 | -0.025 |
| Democratic voters | -0.381 | 0.620 | 0.400 | 0.073 | -0.126 | 0.122 | 0.050 | 0.014 | 0.187 | -0.231 | -0.082 | -0.093 | -0.104 | -0.005 |
| Hypertension | 0.837 | 0.204 | -0.064 | -0.020 | -0.163 | -0.016 | -0.171 | -0.068 | 0.017 | 0.167 | 0.146 | 0.046 | 0.117 | 0.030 |
| Cardiovascular diseases (CVD) | 0.948 | -0.073 | -0.089 | 0.013 | 0.072 | 0.051 | -0.047 | -0.048 | 0.001 | -0.010 | 0.056 | 0.027 | 0.004 | -0.032 |
| Stroke | 0.912 | 0.274 | -0.065 | 0.015 | -0.008 | 0.041 | -0.031 | -0.056 | 0.019 | 0.026 | 0.091 | 0.043 | 0.008 | -0.004 |
| Mental Health | 0.895 | -0.195 | -0.098 | 0.130 | -0.068 | 0.047 | -0.062 | -0.034 | 0.094 | -0.058 | 0.076 | 0.043 | 0.040 | 0.063 |
| Asthma | 0.585 | -0.192 | 0.035 | 0.307 | 0.012 | 0.119 | -0.224 | -0.053 | 0.352 | -0.344 | -0.010 | 0.025 | -0.087 | 0.134 |
| HIV | 0.127 | 0.706 | 0.148 | -0.014 | -0.208 | 0.432 | 0.064 | -0.017 | 0.006 | 0.048 | 0.040 | 0.057 | 0.093 | 0.024 |
| Diabetes | 0.804 | 0.426 | -0.037 | -0.224 | -0.038 | -0.003 | -0.030 | 0.070 | -0.128 | 0.136 | -0.041 | -0.015 | 0.001 | -0.054 |
| Depression | 0.585 | -0.568 | 0.021 | 0.176 | -0.115 | 0.020 | -0.094 | -0.063 | 0.117 | -0.103 | 0.221 | -0.013 | 0.035 | 0.106 |
| Religious affiliation | 0.094 | 0.065 | 0.180 | -0.118 | 0.152 | 0.020 | -0.122 | 0.042 | 0.007 | 0.721 | 0.027 | -0.022 | 0.024 | 0.087 |
| Alcohol | -0.627 | 0.011 | -0.133 | 0.422 | 0.211 | 0.069 | -0.016 | -0.087 | -0.081 | 0.003 | 0.129 | 0.057 | -0.140 | -0.099 |
| Physical inactivity | 0.860 | 0.091 | -0.134 | -0.122 | 0.036 | 0.067 | -0.162 | -0.002 | -0.076 | 0.159 | -0.023 | 0.039 | 0.024 | -0.042 |
| Obesity | 0.718 | 0.078 | -0.180 | -0.001 | 0.149 | -0.122 | -0.408 | 0.001 | -0.085 | 0.121 | 0.072 | 0.061 | -0.043 | -0.002 |
| Low Birth Weight | 0.580 | 0.490 | 0.057 | 0.117 | -0.238 | -0.036 | 0.044 | -0.118 | 0.128 | 0.172 | 0.061 | 0.047 | 0.127 | 0.127 |
| Social associations | -0.022 | -0.121 | 0.059 | 0.307 | 0.356 | 0.056 | -0.213 | -0.190 | 0.321 | 0.385 | 0.292 | 0.035 | -0.168 | 0.015 |
| Cancer | 0.060 | -0.671 | -0.025 | 0.359 | -0.084 | -0.011 | -0.225 | -0.248 | 0.234 | -0.133 | 0.255 | 0.082 | 0.028 | 0.091 |
| COVID vaccination rates | -0.550 | 0.254 | 0.299 | 0.104 | -0.098 | 0.021 | 0.167 | 0.036 | 0.125 | -0.164 | -0.275 | -0.140 | -0.025 | -0.129 |
| Access to Parks | -0.443 | 0.167 | 0.354 | -0.090 | 0.130 | 0.144 | 0.301 | 0.134 | 0.180 | -0.263 | -0.114 | -0.105 | 0.035 | 0.139 |
| Recreation facilities | -0.533 | -0.034 | 0.193 | 0.052 | 0.185 | 0.147 | 0.360 | -0.207 | 0.272 | 0.039 | 0.097 | 0.020 | -0.072 | 0.003 |
| Food environment index | -0.791 | -0.093 | -0.081 | -0.007 | -0.152 | 0.104 | -0.178 | 0.010 | 0.113 | 0.038 | 0.020 | 0.024 | 0.053 | -0.067 |
| Workplace mobility change | 0.388 | -0.278 | -0.305 | 0.099 | 0.260 | -0.087 | 0.031 | -0.135 | -0.004 | -0.006 | 0.160 | 0.189 | -0.082 | -0.034 |
| School | 0.021 | -0.187 | -0.141 | 0.080 | 0.706 | 0.052 | -0.082 | 0.006 | -0.065 | 0.052 | -0.036 | -0.094 | -0.134 | -0.070 |
| Liquor Store Density | -0.265 | 0.121 | 0.055 | 0.013 | 0.042 | 0.032 | 0.024 | -0.038 | 0.677 | 0.045 | -0.094 | 0.043 | 0.056 | -0.110 |
| Grocery and pharmacy mobility change | 0.139 | -0.182 | -0.217 | 0.164 | -0.024 | -0.072 | 0.130 | -0.169 | -0.035 | -0.045 | 0.251 | 0.388 | 0.021 | -0.060 |
| Natural Amenities Scale | -0.050 | 0.110 | 0.055 | -0.159 | -0.046 | -0.088 | 0.794 | 0.219 | -0.030 | -0.159 | -0.125 | -0.036 | 0.039 | 0.033 |
| Environmental hazards | 0.007 | 0.111 | -0.014 | 0.013 | -0.018 | 0.060 | 0.015 | 0.032 | -0.066 | 0.088 | 0.018 | -0.050 | -0.024 | 0.813 |
| Rural population | 0.361 | -0.402 | -0.502 | 0.228 | 0.156 | 0.007 | 0.059 | -0.207 | -0.007 | 0.030 | 0.057 | 0.067 | -0.039 | -0.181 |
| Particulate matter days | 0.096 | 0.065 | -0.002 | -0.046 | -0.105 | 0.062 | -0.048 | 0.806 | -0.146 | 0.075 | 0.237 | -0.037 | -0.016 | -0.041 |
| Ozone days | -0.155 | 0.128 | -0.001 | -0.024 | -0.020 | -0.005 | 0.190 | 0.742 | 0.075 | -0.050 | -0.098 | 0.017 | -0.015 | 0.061 |
| Violent crime | 0.310 | 0.590 | 0.197 | 0.005 | -0.071 | 0.091 | 0.091 | 0.094 | 0.048 | 0.043 | 0.136 | -0.018 | -0.044 | 0.117 |
| Number of primary care physicians | -0.329 | 0.090 | 0.839 | 0.128 | -0.012 | 0.078 | 0.064 | -0.036 | 0.034 | 0.054 | 0.071 | -0.012 | 0.021 | -0.070 |
| Number of internal MDs | -0.291 | 0.168 | 0.811 | 0.096 | -0.100 | 0.161 | 0.052 | -0.018 | -0.026 | 0.070 | 0.065 | -0.024 | 0.022 | -0.086 |
| Hospitals | 0.227 | -0.026 | 0.067 | 0.132 | 0.632 | -0.078 | 0.011 | -0.095 | 0.046 | 0.243 | -0.003 | 0.140 | 0.123 | 0.102 |
| Pharmacies | 0.402 | -0.105 | 0.203 | 0.140 | 0.027 | 0.183 | 0.120 | -0.107 | 0.292 | 0.280 | 0.055 | 0.075 | 0.364 | -0.001 |
| Nursing homes admissions | 0.010 | 0.006 | 0.056 | -0.020 | 0.021 | 0.036 | -0.058 | 0.040 | 0.044 | -0.011 | -0.075 | 0.839 | -0.073 | -0.047 |
| Pediatrics | -0.276 | 0.222 | 0.769 | 0.021 | -0.190 | 0.137 | 0.024 | 0.002 | -0.027 | 0.058 | 0.013 | -0.035 | 0.038 | -0.075 |
| Emergency departments visits | 0.223 | 0.035 | 0.490 | 0.026 | 0.269 | -0.035 | -0.037 | -0.066 | 0.080 | 0.044 | -0.067 | 0.230 | 0.077 | 0.191 |
| ICU beds | 0.100 | 0.180 | 0.531 | 0.102 | 0.247 | 0.007 | -0.087 | -0.038 | 0.059 | 0.171 | -0.059 | 0.461 | 0.165 | 0.114 |
| Mobile van sites | -0.029 | 0.209 | 0.113 | 0.032 | 0.151 | 0.110 | -0.032 | -0.067 | -0.172 | -0.300 | 0.350 | -0.046 | 0.120 | -0.053 |
| Mental health centers | 0.047 | 0.010 | 0.043 | 0.020 | 0.080 | 0.027 | 0.007 | -0.006 | 0.007 | -0.020 | 0.067 | -0.043 | 0.781 | -0.019 |
| Telehealth service provided by hospitals | -0.041 | -0.121 | 0.114 | -0.080 | 0.498 | -0.043 | 0.026 | -0.075 | 0.229 | -0.013 | 0.133 | 0.082 | 0.170 | 0.031 |
| Medically Underserved Areas/population | 0.513 | 0.094 | -0.242 | 0.043 | -0.146 | -0.050 | 0.395 | -0.136 | -0.122 | 0.103 | 0.063 | -0.003 | 0.064 | -0.054 |
| Below poverty | 0.841 | 0.254 | 0.150 | 0.029 | 0.153 | 0.081 | 0.129 | -0.007 | 0.005 | -0.088 | -0.031 | 0.002 | -0.096 | -0.008 |
| Unemployment | 0.782 | 0.134 | -0.176 | 0.155 | 0.008 | 0.023 | 0.273 | 0.089 | -0.136 | -0.045 | -0.115 | -0.008 | 0.135 | -0.100 |
| Median income | -0.826 | -0.001 | 0.048 | -0.162 | -0.295 | -0.013 | -0.072 | 0.098 | -0.039 | 0.029 | -0.090 | -0.055 | 0.138 | -0.016 |
| Income Inequality | 0.353 | 0.288 | 0.445 | 0.137 | -0.071 | 0.215 | 0.388 | 0.001 | 0.049 | 0.103 | 0.063 | -0.032 | -0.088 | -0.107 |
| Population growth | -0.380 | 0.017 | 0.124 | -0.437 | -0.356 | -0.127 | 0.223 | -0.037 | -0.370 | -0.019 | 0.117 | 0.045 | 0.087 | 0.061 |
| Health insurance | 0.428 | 0.175 | -0.090 | -0.490 | 0.101 | 0.005 | 0.259 | -0.097 | -0.295 | 0.196 | 0.070 | 0.026 | -0.114 | -0.099 |
| Renter | 0.123 | 0.490 | 0.526 | -0.240 | 0.116 | 0.286 | 0.023 | 0.096 | 0.073 | -0.162 | 0.028 | -0.018 | -0.064 | 0.124 |
| Married population | -0.320 | -0.639 | -0.346 | -0.116 | -0.136 | -0.159 | 0.011 | 0.026 | -0.101 | 0.252 | -0.007 | -0.010 | 0.096 | -0.039 |
| Gender | 0.095 | 0.012 | 0.353 | 0.059 | -0.485 | 0.045 | -0.059 | -0.036 | 0.258 | 0.232 | 0.096 | 0.027 | -0.165 | 0.158 |
| Race - Non-white | 0.185 | 0.830 | 0.148 | -0.135 | -0.213 | 0.082 | 0.016 | 0.051 | -0.006 | 0.026 | -0.010 | -0.005 | 0.034 | 0.034 |
| Language/ability to speak English | 0.155 | 0.177 | 0.015 | -0.478 | -0.037 | 0.125 | 0.054 | 0.104 | 0.164 | 0.130 | 0.144 | -0.069 | -0.028 | -0.283 |
| Female Headed Households | 0.581 | 0.641 | 0.073 | -0.081 | -0.161 | 0.036 | -0.108 | 0.103 | 0.026 | 0.035 | -0.099 | 0.040 | 0.063 | 0.019 |
| Households with children | -0.165 | 0.023 | -0.028 | -0.574 | -0.103 | -0.166 | -0.355 | 0.278 | -0.293 | 0.095 | -0.191 | -0.061 | 0.172 | 0.000 |
| Educational attainment - college | 0.690 | 0.184 | -0.165 | -0.305 | 0.121 | 0.135 | 0.102 | 0.169 | -0.174 | 0.123 | -0.142 | -0.051 | 0.006 | -0.187 |
| Healthcare related occupation | 0.209 | -0.058 | 0.288 | 0.642 | 0.049 | -0.025 | -0.083 | 0.060 | -0.023 | 0.055 | -0.129 | 0.002 | 0.120 | -0.117 |
| Population density | -0.142 | 0.180 | 0.169 | -0.073 | -0.108 | 0.866 | 0.016 | 0.044 | -0.017 | 0.024 | -0.077 | -0.012 | 0.076 | 0.055 |
| Population with disability | 0.734 | -0.210 | -0.153 | 0.336 | 0.067 | 0.004 | 0.192 | -0.046 | 0.048 | -0.050 | 0.001 | 0.040 | 0.091 | 0.010 |
| Housing Units with No Car | 0.195 | 0.280 | 0.224 | 0.088 | 0.098 | 0.827 | -0.075 | 0.014 | 0.112 | -0.039 | -0.133 | 0.020 | -0.033 | 0.005 |

1. Extraction Method: Principal Component Analysis. Rotation Method: Varimax with Kaiser Normalization.
2. Factor 1- Comorbidities and Social Status, Factor 2- Race and Chronic Diseases, Factor 3- Healthcare Providers, Factor 4- Household Occupation and Children, Factor 5- School and Healthcare Access, Factor 6- Urbanism, Factor 7- Natural Amenity, Factor 8- Air Quality, Factor 9- Liquor Stores, Factor 10- Religions, Factor 11- Policies, Factor 12-Nursing Homes, Factor 13- Mental Health Centers, and Factor 14- Environmental Hazards.
